# Utilising low-cost, easy-to-use microscopy techniques for early peritonitis infection screening in peritoneal dialysis patients

**DOI:** 10.1038/s41598-022-18380-9

**Published:** 2022-08-18

**Authors:** Mark Buckup, Janelle M. Kaneda, Alisha M. Birk, Eleanor Glockner, Ross Venook, Aditya Jain, Shuchita Sharma, Cynthia Wong, Ken Sutha

**Affiliations:** 1 Department of Bioengineering, Stanford University, Palo Alto, CA USA; 2 Department of Immunology, Icahn Institute of Medicine at Mount Sinai, New York City, NY USA; 3 Department of Nephrology, The Mount Sinai Hospital, New York City, NY USA; 4 Department of Pediatrics, Division of Nephrology, Lucile Packard Children’s Hospital, Stanford University, Palo Alto, CA USA

**Keywords:** Transmission light microscopy, Biomedical engineering, Peritoneal dialysis, Computational science

## Abstract

Peritoneal dialysis (PD) patients are at high risk for peritonitis, an infection of the peritoneum that affects 13% of PD users annually. Relying on subjective peritonitis symptoms results in delayed treatment, leading to high hospitalisation costs, peritoneal scarring, and premature transition to haemodialysis. We have developed and tested a low-cost, easy-to-use technology that uses microscopy and image analysis to screen for peritonitis across the effluent drain tube. Compared to other technologies, our prototype is made from off-the-shelf, low-cost materials. It can be set up quickly and key stakeholders believe it can improve the overall PD experience. We demonstrate that our prototype classifies infection-indicating and healthy white blood cell levels in clinically collected patient effluent with 94% accuracy. Integration of our technology into PD setups as a screening tool for peritonitis would enable earlier physician notification, allowing for prompt diagnosis and treatment to prevent hospitalisations, reduce scarring, and increase PD longevity. Our findings demonstrate the versatility of microscopy and image analysis for infection screening and are a proof of principle for their future applications in health care.

## Introduction

Dialysis is the artificial removal of waste products that kidneys usually filter from the blood, and peritoneal dialysis (PD) is an at-home dialysis method that uses the patient's peritoneum to filter out these toxins. Over 26,500 patients in the U.S. and 195,000 patients worldwide use PD to manage their end-stage renal disease^1^. Although haemodialysis (HD) is more common, PD has several advantages, including a survival rate of ten years compared to five years with HD^2^, as well as a more frequent filtration process that frees patients from costly dialysis centre visits multiple times a week, providing significantly higher quality of life than HD^2^.

However, PD leads to a higher risk of peritoneal infection, called peritonitis, which affects nearly 13% of PD users annually and is the leading cause for approximately 16% of PD patient mortality^3^. Severe infection can lead to hospitalisations, which cost approximately $100 million annually in the U.S., or $3800 per PD patient per year^1,4,5^. If left untreated, peritonitis can lead to long-term peritoneal scarring^3^. Excessive scarring prevents the peritoneum from functioning as an exchange barrier, rendering PD impossible and requiring patients to switch to HD^2–4,6^. PD could optimally be utilised for up to ten years, but on average it is used for two to three years before patients prematurely switch to HD due to repeated episodes of infection^3,7^.

Currently, the only preventative measures for peritonitis are standard aseptic techniques, with responsibility falling heavily on patients and caretakers to mitigate risk. When a patient presents with peritonitis signs and symptoms such as cloudy effluent or abdominal pain, physicians collect an effluent fluid sample for a cell count and culture to confirm peritonitis diagnosis. If the white blood cell (WBC) count is > 100 cells/mm^3^ and 50% of those cells are responding neutrophils, antibiotics and antifungal prophylaxis are started immediately while waiting for results from a bacterial culture, antibiotic sensitivity test, and Gram stain^3^. However, reliance on patient-reported, subjective symptoms results in a delay from the onset of clinically meaningful infection to the treatment of peritonitis. One study demonstrated that by the time patients start antibiotics, their effluent WBC concentration is often 2250 cells/mm^3^, well beyond the 100 cells/mm^3^ benchmark^8^. This extended time to diagnosis and treatment from initial infection increases peritoneal scarring. As the number of kidney disease patients rises around the world^9^, and with the push for home dialysis from the 2019 “Advancing American Kidney Health” executive order^10^, at-home dialysis methods like PD are critical areas for innovation.

With the increased versatility and ease of use of inexpensive microscopy systems along with the expanding frontier of image analysis technologies^11–13^, we created an in-line optical method for accurate WBC screening to address this problem, guided by three overall aims. First, the solution would need to be inexpensive enough to have clear economic value for at-home use. Then, it would need to be specific against solutes and debris found in effluent, such as red blood cells, salts, and triglycerides^14,15^, which could confound WBC predictions in a patient-specific way. Lastly, it would need to be user-compatible.

Our prototype, named OpticLine, uses microscopy and image analysis technology to detect peritonitis before patients notice symptoms. By serving as a screening device to notify patients as soon as their WBC count reaches levels indicative of infection, OpticLine allows nephrologists to provide treatment earlier, reducing acute hospitalisation costs and preventing peritoneal scarring to improve PD longevity. WBC quantification enables early detection of peritonitis from multiple causes, including fungal, viral, and bacterial infections. While haematology analysers have been proposed as a viable method for detecting WBCs in bodily fluids^16,17^, we demonstrate the first application of microscopy to screen for peritonitis. Our technology can be constructed from inexpensive, off-the-shelf components, offering an affordable way to prevent peritonitis progression and improve patient outcomes from home. With OpticLine, we demonstrate accurate and consistent WBC quantification in benchtop and simulated continuous cycling PD setups for screening, while also accounting for potential solute confounders. Lastly, our market research and human factors studies demonstrate eagerness for routine incorporation, ease of use, and device favorability among key stakeholders.

## Results

### Inexpensive design and simple insertion into drain line

**Figure 1:**
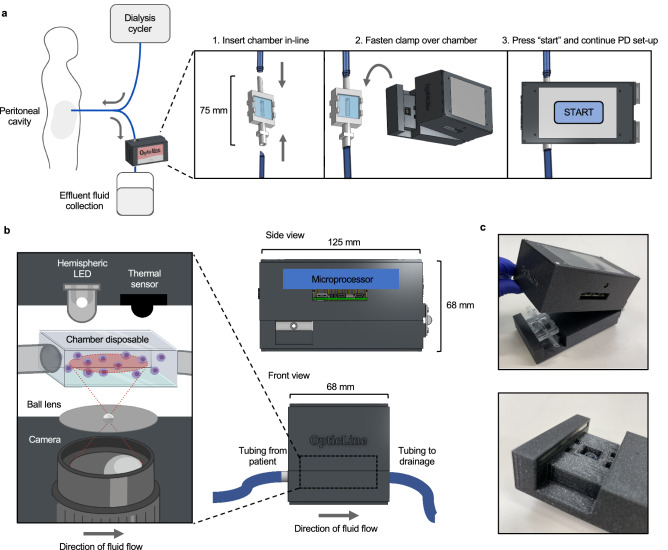
Overview of OpticLine use and mechanical construction. (**a**) The OpticLine chamber is inserted in-line with the drainage tubing, between the drain line and effluent fluid collection, when the patient is setting up for their peritoneal dialysis (PD) session. The OpticLine clamp then clamps over the chamber and images effluent as it flows through the clamp during drain times. The user interacts with the device via a touchscreen on the top of the clamp, and presses “Start” when they begin their PD session. Created with Onshape.com. (**b**) As labelled on front and side views, the clamp is 125 mm long, 68 mm wide, and 68 mm tall. The top part of the clamp contains the microprocessor. The expanded view on the left shows the clamp’s optical components in relation to the viewing chamber. The top part of the clamp contains a hemispheric LED that provides backlight for the lens, and a thermal sensor that detects fluid flow through the chamber and signals to the microprocessor that the camera should begin capturing images. The bottom part of the clamp contains the camera, which is connected to the microprocessor, and a ball lens that magnifies the camera’s view 140×. Created with Onshape.com and BioRender.com. (**c**) Photographs of an OpticLine prototype. The top photo shows the entire clamp with the chamber inserted. The bottom photo is a close-up of the clamp’s optical interface. The microscope lens is recessed in the square hole, and the four small rectangular divots mate with prongs on the chamber to prevent it from sliding during use.

OpticLine consists of two parts: (1) a reusable imaging unit that clamps over (2) a disposable fluid flow chamber that is inserted between the PD drain line and effluent fluid collection (Fig. 1a).

The imaging unit, or “clamp,” houses all circuitry and is clamped over the chamber to align the optical hardware with the chamber’s viewing window. It is driven by a Raspberry Pi 4 (4 GB RAM), which runs a Raspbian Linux operating system and is powered by a 120–240 V AC supply. A printed circuit board was custom-designed using Fritzing and mounted directly on top of the Pi. This printed circuit board houses OpticLine’s electrical components and connects to the input/output pins of the Pi below it. Data is stored on the on-board SD card. A 3.5″ Nextion touchscreen display communicates with the Pi through its serial ports, through which the patient interacts with. The clamp contains an 8 MP camera mounted to a 2 mm borosilicate glass ball lens in a plastic aperture, providing a refractive index of 1.51 and magnification of 140× with 2 μm resolution^18^. This allows OpticLine to capture a field of view of approximately 1000 × 1000 μm (Supplementary Fig. 1). The camera is connected via a 300 mm flex cable directly to the Pi. A hemispheric LED is positioned opposite of the chamber, camera, and ball lens, offset by 1 mm in *x* and *y* directions to cast shadows on cells flowing in the chamber for improving visualisation (Fig. 1b). The camera backlight is driven by the 5 V power supply and provides roughly 6000 mcd of optical brightness approximately 20 mm above the disposable chamber. This design provides both direct and ambient light for better illumination when imaging the flowing effluent. A thermal sensor is connected in series with an N-MOSFET. The thermal sensor is used to detect the presence of flowing effluent, indicating that our device should begin capturing images. Photos are captured for the first 30–60 s of what is typically a 5-min period of constant flow followed by 5–10 min of pulsatile flow. OpticLine captures images at a back focal distance of 0.56 mm within the viewing chamber^18^, through which WBCs in effluent flow after draining out of the patient’s peritoneal cavity. OpticLine’s camera has an exposure time of 10 μs to ensure images of fast-moving cells are in focus.

The disposable chamber is intended to be single-use and replaced every session to avoid potential loss of accuracy due to sediment buildup on the viewing window, although we confirmed no change in performance over ten days of continuous use (Supplementary Note 1 and Supplementary Fig. 2). Replicated male and female connector counterparts were designed to fit securely with the corresponding connectors in the native PD tubing (Fig. 1c). Two standard glass coverslips are attached on either side of the chamber, creating a viewing window that allows the camera to image the flowing effluent. The chamber’s inner channel maintains the same cross-sectional area as the drain line tubing, preventing any backflow pressure and maintaining the effluent’s drainage speed as it leaves the peritoneal cavity. By using exclusively off-the-shelf processes and components, OpticLine and future devices utilising our imaging technology can be inexpensively created for approximately $240 USD (Supplementary Table 1, 2).

An alternative approach to implement our microscopy technology is to use a compound rather than a ball lens. Despite increased size and cost, preliminary experimentation has demonstrated that this lens can image directly across parts of PD tubing sets without an in-line disposable chamber (Supplementary Note 2 and Supplementary Fig. 3).

### Computational pipeline and dialysis workflow integration

**Figure 2:**
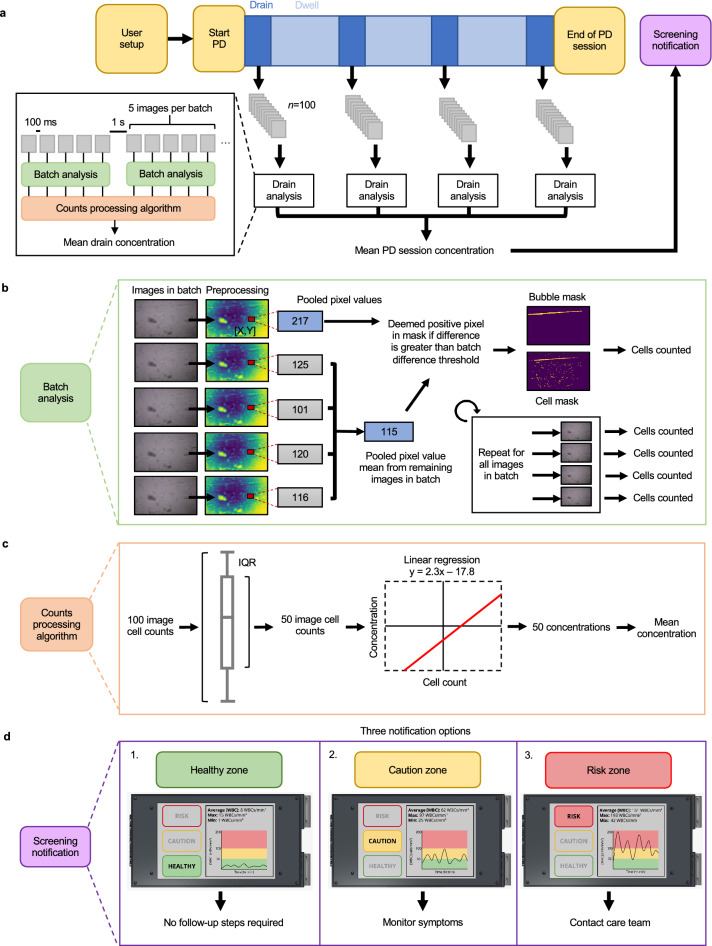
Computational design. (**a**) During a peritoneal dialysis (PD) session, OpticLine captures 100 images during each drain in batches of 5 spaced 100 ms apart, with a gap of 1 s between batches. Each batch is independently analysed and fed into the counts processing algorithm, which calculates the mean white blood cell (WBC) concentration for each drain. These mean drain concentrations are then averaged to a final mean PD session concentration. This concentration, along with a “healthy, caution, or risk” notification is reported to the patient at the end of their PD session. (**b**) The images in each batch are preprocessed with a series of filters and blurs. The value of each pixel in each batch image is evaluated against the average value of the pooled pixels at the same (*x*, *y*) coordinates in the other four images of the batch. If the difference between the value of one pixel in an image and the average value of the pooled pixels at that location in the other four images is greater than the defined batch difference threshold, that pixel is marked as positive in the cell mask. This mask is analysed to calculate the number of cells. (**c**) Our algorithm takes the interquartile range (IQR) of the cell counts for each set of 100 images to eliminate outliers, leaving 50 cell counts for each drain. These 50 counts are then converted to concentrations with the linear regression model developed during algorithm training. These 50 concentrations are then averaged to report the mean WBC concentration for the drain. (**d**) Example screen displays. At the end of a PD session, OpticLine displays a graph of the patient’s WBC concentration throughout the duration of the session; the average, maximum, and minimum WBC concentration for the session; and a notification reporting whether the session’s mean WBC concentration falls in the healthy, caution, or risk zone. If the concentration is healthy, no follow-up steps are required; if in the caution zone, patients are advised to monitor their symptoms; if in the high-risk zone, the patient is recommended to contact their care team immediately for further testing.

OpticLine captures images during each draining interval of the user’s PD session, excluding the priming drain step (Fig. 2a). The fluid detection module is triggered 5–10 s after flow begins, followed by 30–60 s of image capture, all within the 10–15 min of average drainage time^19^. One hundred images are captured in batches of five during each drainage interval, and each batch is then analysed by the microprocessor during the dwell periods (Supplementary Fig. 4).

A batch of five images is input into the image batch analysis algorithm, which outputs a cell count for each image (Fig. 2b, Supplementary Note 3, Supplementary Fig. 5, and Supplementary Fig. 6). Each image is cropped to exclude blurry areas outside the focus of the lens, downscaled to reduce noise, and contrasted for maximal contour detection and optimal cell counting. The value of each pixel in the processed image is compared to the mean value of the pooled pixels at the same (*x*, *y*) coordinates in the other four images of the batch. If the difference is greater than a tunable batch difference threshold, the current pixel is marked positive in the cell mask. Potential moving cells are identified by detecting the variation across images in the batch. Gaussian blurs and size thresholds are used to denoise the mask and combine turbulence that is associated with each cell. Similarly, a mask for bubbles and artifacts is determined by downscaling the preprocessed image. A tunable batch difference threshold and size filters were optimised to capture bubbles and moving artifacts. Any cell centroids residing in areas deemed as bubbles or artifacts are removed. With this strategy, we can remove stagnant noise associated with the camera or setup, as well as moving bubbles and other solutes in the effluent fluid that are not consistent across the batch of five images.

The output image cell counts are filtered for outliers and converted to WBC concentrations for each drainage interval through the counts processing algorithm (Fig. 2c). This algorithm predicts effluent concentration from cell count using a trained linear regression model. At the end of the PD session, OpticLine averages all mean drainage concentrations to provide a screening result to the patient (Fig. 2d). An overall mean predicted concentration within 0–50 WBCs/mm^3^ is deemed “healthy,” recommending that the patient continue performing PD. A mean within 50–100 WBCs/mm^3^ is considered “intermediate,” recommending that the patient monitor symptoms closely and contact their care team if they remain in that range for more than a few days. A mean at or above 100 WBCs/mm^3^ is deemed “at risk,” and OpticLine recommends that the patient contact their care team as soon as possible for follow-up diagnostic testing.

### Training and validation of computational pipeline

**Figure 3:**
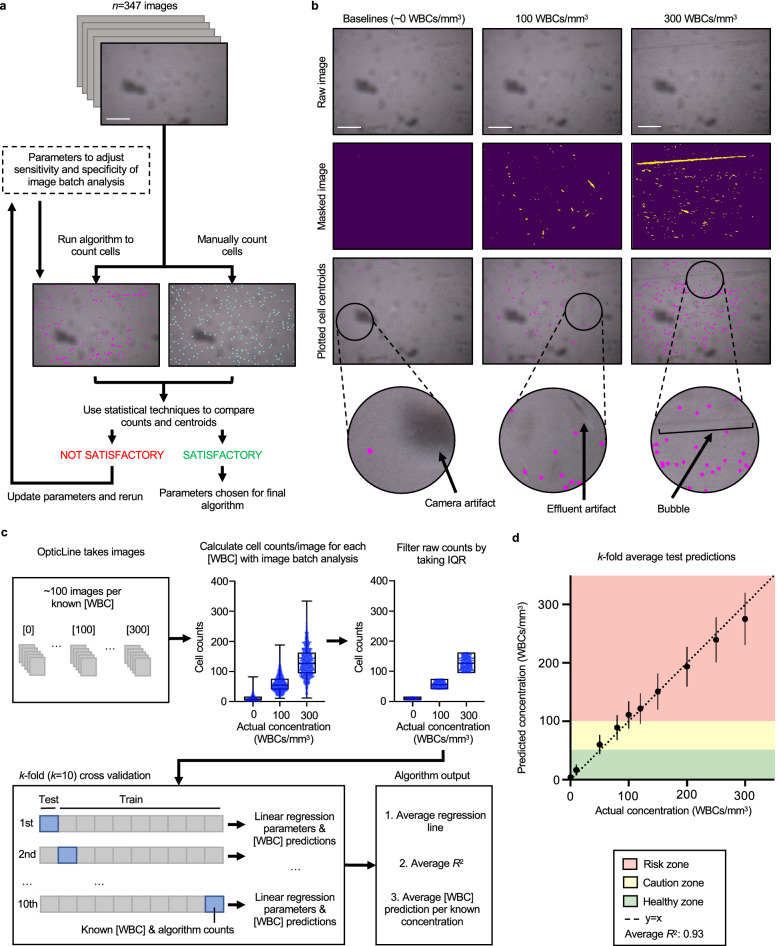
White blood cell counting algorithm training and validation. (**a**) White blood cell (WBC) counts from the image batch analysis algorithm were compared to manual counts from one rater across concentrations 0, 10, 50, 100, 200, and 300 WBCs/mm^3^ to determine optimal parameters for maximising specificity and sensitivity using 347 images taken by OpticLine. No cell staining was used for manual counting. Parameter optimisation was iterative, and the final selected parameters produced algorithm cell counts that had the least significant difference with manual counts, evaluated with Wilcoxon signed-rank tests (with continuity correction) and Pearson correlation coefficients. Scale bar represents 200 μm. (**b**) Algorithm cell centroid outputs using optimal parameters for images of WBCs in patient effluent at concentrations of 0, 100, and 300 WBCs/mm^3^. Raw images (top row) are masked (middle row) and cell centroids are plotted on the raw images (bottom row). Arrows highlight artifacts from the camera and moving effluent. Scale bar represents 200 μm. (**c**) Batches of images (*n* = 100) of each tested WBC concentration were run through the image batch analysis algorithm to output raw cell counts. To limit noise, raw counts were filtered by taking the interquartile range (IQR), only keeping the middle 50% of the counts. *k*-fold cross validation (*k* = 10) was performed on the filtered counts to train and test predictions for WBC concentrations. Within each iteration, 10% of the data was used for testing and the remaining 90% was used for training an ordinary least squares linear regression model. An average regression line, *R*^2^, and WBC concentration predictions per known concentrations are output. (**d**) Graph of the *k*-fold average test predictions across *k* = 10 iterations of the cross validation vs. actual concentrations of the effluent samples. Error bars are s.d. The predicted and actual WBC concentrations were similar, falling close to one-to-one correlation as represented by the *y* = *x* line (dotted line) with average *R*^2^ across the *k* = 10 iterations of 0.93.

OpticLine’s image batch analysis depends on multiple parameter values which can be tuned to optimise sensitivity and specificity (Fig. 3a). These parameters range from Gaussian blurring to eliminate pixelated noise to size thresholds to accurately discern cells from larger artifacts. To find the ideal combination of parameter values to achieve appropriate sensitivity and specificity, we performed the image batch analysis algorithm with 96 combinations of parameter values to output cell counts for 347 images, spanning 6 spiked concentrations ranging from healthy baseline to 300 WBCs/mm^3^ in effluent from 3 patients. We chose the parameter combination set that produced the least significantly different cell counts when compared to those manually counted by one rater (without cell staining), evaluated with Wilcoxon signed-rank tests (with continuity correction) and Pearson correlation coefficients. The manually-counted images were also used to evaluate the performance of the image batch analysis algorithm for cell detection, using the ideal parameter set (Supplementary Note 4 and Supplementary Fig. 7). However, predicted WBC concentration, rather than image cell count, is used for infection screening. The ideal parameter combination set was used in training and testing for predicting WBC concentrations in subsequent experiments (Fig. 3b).

An ordinary least squares linear regression model was trained with *k*-fold (*k* = 10) cross validation to predict WBC concentrations (Fig. 3c). Using *k*-fold cross validation prevents overfitting and maximises data used to train and test a linear regression model^20^. One hundred images of each spiked concentration sample (0, 50, 80, 100, 120, 150, 200, 250, and 300 WBCs/mm^3^) for each of eighteen patient effluent samples were input into the image batch analysis pipeline to obtain numerical cell counts. Image batch analysis counts of the same spiked concentration across multiple patient effluent samples were analysed in aggregate (*n* = 1800). To eliminate outliers, interquartile ranges of the aggregate image batch analysis outputs at each spiked concentration sample were input into the linear regression model (*n* = 900). The WBC concentration predictions averaged across the *k*-fold iterations fall close to a *y* = *x* line (Fig. 3d). The mean *R*^2^, adjusted for heteroskedasticity, was 0.93 (s.d. = 0.0008). There was little variation in regression parameters (s.d. of coefficient = 0.004). Comparisons between algorithm outputs corresponding to 10, 50, and 100 WBCs/mm^3^ were statistically significantly different (*p* < 0.0001).

We also evaluated whether a nested cross-validation approach to train and validate our computational pipeline could improve prediction accuracy (Supplementary Note 5, Supplementary Fig. 8, and Supplementary Table 3). While this approach yielded promising results (*R*^2^ = 0.76), we used the current prediction model that had the best balance between sensitivity and specificity.

### Screening classification with clinically collected effluent

**Figure 4:**
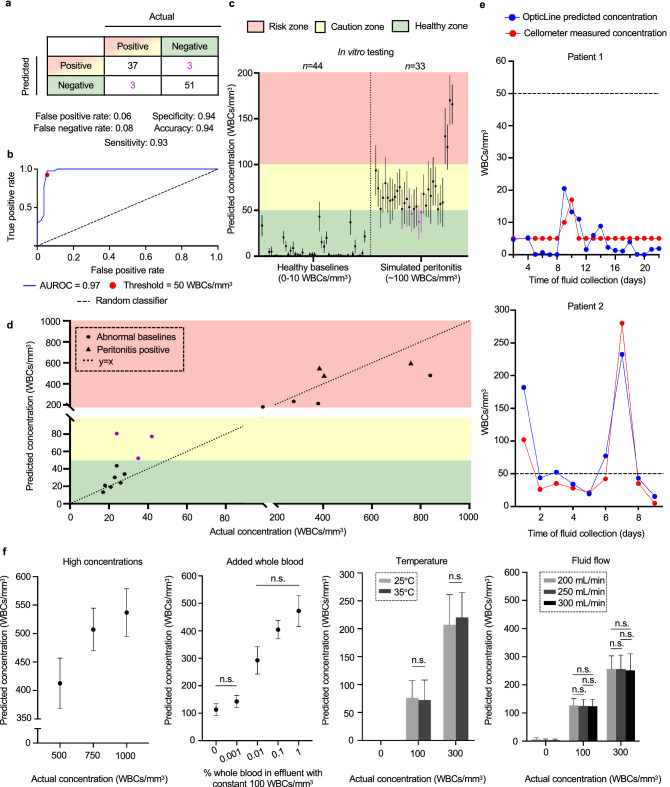
In vitro results using clinically collected patient effluent samples. (**a**) Confusion matrix for 94 peritoneal dialysis patient effluent samples using a 50 white blood cells (WBCs)/mm^3^ threshold separating “positive” for infection caution (yellow, red zones) from “negative” for infection caution (green zone). Some of the healthy baseline samples (0–10 WBCs/mm^3^) were spiked to a concentration of 100 WBCs/mm^3^ (*n* = 33). (**b**) The receiver operating characteristic (ROC) curve, evaluated by varying the binary classification threshold from 0 WBCs/mm^3^ to the maximum WBC concentration prediction in the dataset. The area under the ROC (AUROC) was 0.97. The red marker denotes the threshold balancing sensitivity and specificity (50 WBCs/mm^3^). The dotted line represents the ROC of a random classifier for comparison. (**c**) Predicted WBC concentrations for effluent samples with healthy baseline concentrations (*n* = 44) (left) and those spiked to about 100 WBCs/mm^3^ (*n* = 33) (right). Predicted concentrations were classified in the healthy zone if < 50 WBCs/mm^3^ (green), caution zone if between 50 WBCs/mm^3^ and 100 WBCs/mm^3^ (yellow), and in the risk zone if ≥ 100 WBCs/mm^3^ (red). False negatives are shown as purple markers. Error bars are s.d. (**d**) Predicted WBC concentrations of effluent samples with elevated baseline WBC concentrations above 10 WBCs/mm^3^ (*n* = 17) (circle markers), including three lab-confirmed peritonitis-positive samples (triangle markers). Most samples with concentration greater than 10 WBCs/mm^3^ are correctly classified; three false positives are shown as purple markers. (**e**) Comparison of predicted WBC concentrations in patient effluent collected over time from OpticLine (blue) and a lab-grade Cellometer (red). The dotted line represents the binary classification threshold of 50 WBCs/mm^3^. **f,** Predicted WBC concentrations across different sample levels for four potential confounders. High WBC concentrations were produced by spiking WBCs in patient effluent (left). Percent whole blood was added to effluent spiked to a concentration of 100 WBCs/mm^3^ (left, middle). Temperature (right, middle) and effluent flow rate (right) at sample measurement were also evaluated. For an actual concentration of 0 WBCs/mm^3^, OpticLine predicted concentrations of 0 WBCs/mm^3^ with no s.d. at both tested temperatures. Error bars are s.d. “n.s.” represents no statistically significant difference between the given samples.

The trained linear regression model was used to predict WBC concentrations of 94 PD effluent samples and assess OpticLine’s binary classification. The threshold for infection concern (“positive”) was 50 WBCs/mm^3^. OpticLine warns users to start monitoring fluid closely at this level, while WBC concentration predictions below 50 WBCs/mm^3^ are “negative” for infection concern (Fig. 4a). Binary classification was evaluated using mean predicted WBC concentrations for each sample. OpticLine has 94% accuracy for infection caution screening. We evaluated the discrimination performance of the binary classification using the area under the receiver operating characteristic (AUROC) for all possible infection concern thresholds (Fig. 4b). With a threshold of 50 WBCs/mm^3^, AUROC was 0.97.

To accurately measure effluent fluid concentrations and compare to OpticLine’s concentration predictions, we tested healthy effluent (*n* = 44) and simulated infected effluent (*n* = 33) on both a Cellometer Auto 2000 (Nexcelom Biosciences) and OpticLine (Fig. 4c). To simulate infected effluent, we spiked healthy effluent (0–10 WBCs/mm^3^) with peripheral blood mononuclear cells (PBMCs) to a concentration of 100 WBCs/mm^3^. The majority of our samples (*n* = 29) were spiked with freeze-thawed PBMCs, while the last four simulated samples were spiked with fresh PBMCs within four hours of isolation. We believe that OpticLine undercounted freeze-thawed samples because of the higher number of dead cells, which are smaller and harder to quantify in our setup. True infected effluent would be similar to our freshly-spiked samples, so we are confident that OpticLine is sensitive enough to quantify real-world samples.

We collected 17 effluent samples with naturally elevated WBC concentrations at baseline (> 10 WBCs/mm^3^) to further validate OpticLine’s cell counting abilities (Fig. 4d). Three of seventeen samples were confirmed peritonitis-positive, as determined by overall WBC (> 100 WBCs/mm^3^) and neutrophil (> 50% cells) counts, while fourteen of seventeen samples had elevated counts, likely due to recent PD catheter surgery or retrograde menstruation. All three peritonitis-positive samples were correctly predicted, suggesting clinical validity. OpticLine counts of effluent samples collected over consecutive days for two different patients closely aligned with WBC concentrations both above and below 50 WBCs/mm^3^ determined by the Cellometer (Fig. 4e).

We tested the effects of four potential confounders on our imaging system: (1) high WBC concentrations, (2) whole blood (WB) in effluent, (3) different temperatures, and (4) different effluent flow rates through the device (Fig. 4f). High WBC concentrations (> 300 WBCs/mm^3^) are classified within the risk zone, appropriately alerting the patient to contact their care team, although they were underpredicted because OpticLine was optimised for high sensitivity in the clinically relevant range of 0–300 WBCs/mm^3^. We spiked simulated-infected effluent (100 WBCs/mm^3^) with WB (0.001–1% in effluent) to determine the confounding effects of WB presence, which can occur from retrograde menstruation or ascites^21^. As expected, increased blood resulted in higher WBC predictions, because WB contains both WBCs and red blood cells. However, there was no statistically significant difference between WBC concentration predictions at baseline and 0.001% WB, so only significant amounts (0.01% and greater) would potentially confound OpticLine’s predictions.

To evaluate the effect of temperature and flow rate, we ran effluent samples with 0, 100, and 300 WBCs/mm^3^ at physiological temperatures of 25 and 35 °C and PD cycler flow speeds of 200, 250, and 300 mL/min^22^. We tested 25 and 35 °C to mimic fluid temperature exiting the peritoneum cavity, which is around 37.5 °C, and to account for heat loss from effluent travelling through tubing before reaching OpticLine. Temperature did not confound concentration predictions at 0, 100, and 300 WBCs/mm^3^. As flow rate increased, mean WBC concentration prediction decreased slightly, as greater flow created more turbulence in the viewing window. However, there were no statistically significant differences between flow rates for WBC predictions of 100 and 300 WBCs/mm^3^ (Fig. 4f and Supplementary Fig. 9).

### User feedback in market research and human factors studies

**Figure 5:**
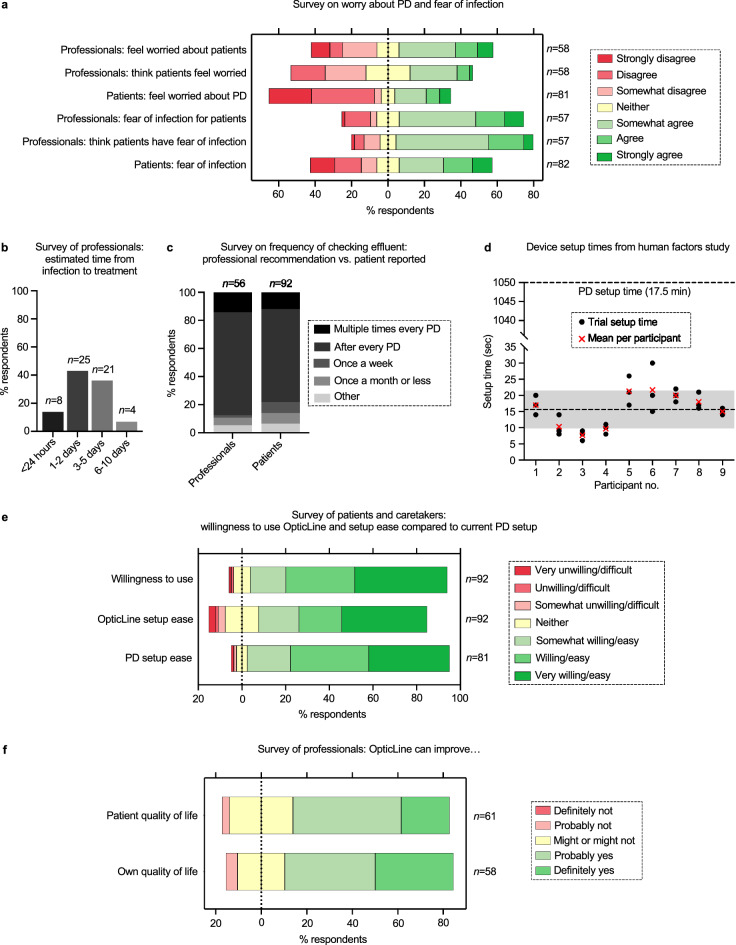
Market research and human factors study results. Percentage of respondents shown on the *x*-axis (**a**, **e**, **f**) or *y*-axis (**b**, **c**) and number of respondents (*n*) shown on the right *y*-axis (**a**, **e**, **f**) or above each response bar (**b**, **c**). (**a**) Seven-point Likert scale results comparing opinions from professionals and current and past patients and caretakers on worry about peritoneal dialysis (PD) (top three), and fear of peritonitis (bottom three). Professionals were asked if they feel worried about patients and if they think patients feel worried right before, during, or right after a PD session. Similarly, professionals were asked if they are afraid for their patients and if they think their patients are afraid of potential infection when they set up or do PD. (**b**) Professional respondents who see patients selected time bins (*x*-axis) for estimated duration of a five-step process from peritoneal infection to treatment. Dark to light grey bars (left to right) represent shortest to longest time options, respectively. (**c**) Reported recommendation for frequency of chequeing effluent from professionals who see patients versus frequency reported by current and past patients and caretakers from both studies. Dark to light grey sections (top to bottom) represent highest to lowest frequency, respectively. (**d**) Device setup times (s) from the human factors study for nine participants. Black circles represent three individual trial times per participant. Red “x” markers represent mean setup time among trials for each participant. Mean setup time across all trials and participants (15.6 s) shown with bottom dotted line; grey bar represents ± s.d. (5.9 s). This compares to the average PD setup time of 1050 s or 17.5 min (top dotted line). (**e**) Seven-point Likert scale responses from past, current, and prospective patients and caretakers on willingness to use OpticLine (top bar), how difficult they think it would be to set up OpticLine (middle bar), and for current and past patients and caretakers, the difficulty of their PD setup (bottom bar). (**f**) Professional respondents ranked on a five-point Likert scale for thinking the device could significantly improve PD patients’ quality of life and their own quality of life.

We administered an anonymous online market research questionnaire to past, present and prospective PD patients and caretakers, and PD professionals (nephrologists, nurses, and researchers) to gather insight from key stakeholders and connect PD experience with device feedback (Fig. 5). We received 153 responses in total, including from 61 professionals, and 50 current and 29 past PD patients (Supplementary Table 4). Most respondents were 45 to 64 years old, female, and White, living across five geographic regions in the U.S. (Supplementary Table 5, 6). About 54% of current and past patients have been on PD for 2–5 years (Supplementary Table 7). About 65% and 81% of respondents said the questionnaire was very clear and easy to complete, respectively (Supplementary Fig. 10a).

Of the professional respondents who see patients (*n* = 58), about 52% worry about their patients doing PD and 35% think their patients feel worried, while only 31% of current and past patients report feeling worried (Fig. 5a). About 68% of professionals reported fear of potential infection (peritonitis) for their patients and 76% reported that they think their patients are afraid, while 51% of patients reported being afraid (Fig. 5a).

Most professionals estimate the time from infection to treatment as 1–5 days (Fig. 5b). About 73% of professionals tell their patients they should cheque their effluent for cloudiness and signs of infection after every PD session, and 66% of current and past patients and caretakers report this frequency (Fig. 5c). Although there appears to be a standard for chequeing effluent for signs of infection, estimated time from infection to treatment varies.

We designed a human factors study recruiting paediatric caretakers to collect device setup times and user feedback for OpticLine. Participants engaged in a short introductory Zoom meeting either before their appointment or after clinical care with at least one study team member, where the participant was shown a short video demonstrating OpticLine’s setup, told a brief study background and motivation, and invited to ask any questions before deciding to participate. Informed consent and assent were then obtained at the dialysis clinic. The participant could watch the setup video again, ask any questions, and practise as much as they liked before beginning the setup trials. The prototype was 3D-printed, non-functioning, and the same weight as a fully functional device. Setup times were recorded for three trials, with timing beginning after the removal of the tubing caps and finishing when the clamp was securely fastened over the viewing chamber. Nine participants were timed for three device setup trials, and eight of the participants completed a six-item questionnaire.

Most of the caretakers’ patients were 10–18 years old and Hispanic. Two patients had at least one hospitalisation for peritonitis, and three had at least one hospitalisation for non-peritonitis PD complications (Supplementary Table 8). The mean setup time across all participants and trials was 15.6 s, with four of the intra-participant mean times falling below this value (Fig. 5d). Times tended to decrease across subsequent trials. OpticLine setup time is insignificant when considering an average PD setup time of 17.5 min (Fig. 5d and Supplementary Note 6). The participants who completed the follow-up questionnaire ranked the device as very easy to set up.

Over 90% of past, current, and prospective patients and caretakers in the market research study reported willingness to use OpticLine, and 77% thought OpticLine would be easy to set up, similar to the ranked difficulty of the PD setups for current and past users (Fig. 5e). Over half of all patients and caretakers reported that they think OpticLine would improve their experience with PD (Supplementary Fig. 10b). About 69% and 74% of all professionals believe OpticLine can significantly improve patient quality of life and their own quality of life as a professional working with PD, respectively (Fig. 5f). About 98% of all professionals would or would maybe be in favour of adopting the device into standard clinical practise for PD and recommend the device to their patients (Supplementary Fig. 10c).

## Discussion

We have designed, built, and validated a technique that screens for peritonitis with high accuracy before perceptible infection symptoms by using in-house white blood cell quantification algorithms to analyse microscopic images of patient effluent. Although there are other devices currently on the market for peritonitis detection^23,24^, our application of microscopy is unique for its inexpensive construction, focus on white blood cell detection, and simplistic in-line design that monitors peritoneal dialysis sessions in real time, an advantage that will increase patient adoption as demonstrated by our user studies. Additionally, while our findings emphasise the impact of imaging modalities in dialysis, they also allude to a broader application of microscopy and its ability to impact interdisciplinary fields in medicine and health care.

Although automated microscopy-based methods for counting WBCs in bodily fluids are known to overestimate WBC counts and have decreased accuracy at higher concentrations of WBCs^16,17,25^, we found that our method tended to underpredict WBC concentrations greater than baseline levels. Because OpticLine is a screening device, we set the infection classification threshold at 50 WBCs/mm^3^ to minimise false negative rates and prioritise sensitivity. Microscopy-based methods can also decrease in accuracy in the presence of lipids, red blood cells, protein, and cell debris^25,26^. Cloudy, infected effluent could thus pose a challenge for microscopy-based screening tools. With our technology, we optimised a batch analysis process that can detect artifacts and cell centroids by determining variation among sequential images. We confirmed that fibrin, protein, and other debris do not confound our WBC predictions (Supplementary Note 1 and Supplementary Fig. 2). Although significant amounts of whole blood did confound our predictions, it also tints the effluent pink, alerting the patient to abnormal drainage.

While OpticLine’s unsupervised image analysis to detect turbulence successfully discerns baseline from infection-indicating PD effluent, more advanced, open-source computational methods can be used for better cell detection and segmentation—such as convolutional neural networks—although they must be adapted, since we image flowing fluid as opposed to static biomedical images^27^. More complex supervised learning approaches could be employed during the calibration of OpticLine (Supplementary Note 5, Supplementary Fig. 8, and Supplementary Table 3). Additionally, an extra affine or translational registration step to align the batch of five images can be added to ensure minimal noise during image capture and analysis^28^. While these extra steps may refine cell detection and increase accuracies, they require heavier computational resources and longer run-times. OpticLine uses an off-the-shelf Raspberry Pi microprocessor, and its mechanical design ensures minimal movement between images, in addition to a series of blurs that account for any movement, which are sufficient for our current binary screening strategy (Supplementary Fig. 6). However, a custom-designed chip or GPU-optimised microprocessor^29^ can be utilised for deep learning techniques if our imaging technology is used in future diagnostic applications requiring higher cell detection accuracy.

Currently, many incoming dialysis patients are hesitant to use PD because of the risk of infection^30^. If widely adopted, OpticLine and its technology could increase the attractiveness of PD, which is crucial for fulfilling the 2019 executive order that mandates 80% of new end-stage renal disease patients receive either at-home dialysis or a kidney transplant by 2025^31^. PD is already the dominant dialysis method in many countries around the world^32^, with 95% of PD patients in developing countries^1^. Due to its low cost, screening accuracy, and ease of use, OpticLine is particularly valuable for increasing PD safety in these communities, where lack of standard infection prevention training leads to high peritonitis rates^32^.

Although designed for infection screening, this technology can be further validated in future studies that improve our current WBC detection algorithm by identifying any additional confounding effluent solutes, facilitating more complex concentration prediction model training (Supplementary Note 5, Supplementary Fig. 8, and Supplementary Table 3). With additional clinical validation evaluating trends in WBC concentration throughout a PD session, our application could become a diagnostic tool, expediting and clarifying the time from infection to treatment by allowing clinicians to prescribe definitive treatment (Supplementary Note 7). Patients would also need to visit the clinic less often, improving quality of life. In addition to adapting to other PD modalities, the technology could be used in tandem with other technologies that screen for infection, such as testing strips or spectrophotometry^33^ (Supplementary Note 8 and Supplementary Fig. 11). Multi-modal screening could allow for more specific at-home screening and diagnosis. With the incorporation of a more powerful lens that could image directly across PD tubing, our technology could be incorporated directly into the cycler as well, eliminating all burden on patients and caretakers (Supplementary Note 2 and Supplementary Fig. 3).

The versatility of our imaging application can be used to manage disease progression and screen for infection in other bodily fluids as well. For hospitalised or at-home patients with indwelling urinary catheters^34,35^ and hydrocephalus patients using external ventricular drains, infection is a common complication that is currently predicted by cell count^36,37^ and could potentially be detected with our technology. We hope that our technology’s simplicity and novelty of its microscopy-based image analysis will not only improve the lives of PD patients, caretakers, and physicians, but also highlight and advance imaging-based screening tools in medical care.

## Methods

### Mechanical construction

The clamp body was designed in Onshape and 3D-printed via fused deposition modelling with neodymium magnets added on either side of a compressible gasket to create a positive lock mechanism that fits securely around the chamber. The clamp is 125 mm long, 68 mm wide, 68 mm high, and weighs approximately 300 g. The 2 mm glass ball lens was acquired from a Foldscope^18^ and mounted onto the V2 Raspberry Pi camera via epoxy. The disposable chamber was designed in Onshape and printed via stereolithography. It is made with Accura 60 plastic and has dimensions 77 mm (length) by 28 mm (width) by 12 mm (height). It attaches to the effluent drain line’s existing male and female connectors. The coverslips are standard glass microscopy coverslips, 484 mm^2^ and 0.16 mm thick, which are attached to either side of the printed piece with two-part epoxy.

### Electronics

All electronics are housed inside the clamp. Computational analysis occurs onboard rather than over the cloud because a large percentage of the U.S. PD population resides in rural communities, where fast internet access may be inaccessible^38^.

### Effluent sample collection and image batch analysis training

To develop our computational pipeline, we collaborated with the Nephrology Department at the Icahn Institute of Medicine at Mount Sinai to acquire effluent samples from multiple patients over multiple PD sessions. These samples were collected from PD patients who performed their dialysis either inside or outside of the hospital. All experimental protocols and experiments were approved by the Institutional Review Board of The Mount Sinai School of Medicine (IRB-20-04268) and were performed in accordance with the relevant guidelines and regulations. After informed consent was obtained, all samples were collected 0–8 h after the end of the patient’s PD session from the patient’s effluent drainage bag. We measured the baseline cell count of each sample with both a haemocytometer and a Cellometer Auto 2000 (Nexcelom Biosciences) cell counter. If the sample was deemed “healthy,” containing a baseline cell count of 0–10 WBCs/mm^3^, we took images of that sample. If the sample had a cell count over 10 WBCs/mm^3^, we took baseline images and noted that it was not healthy. Because it was difficult to acquire many peritonitis-positive samples from the hospital, we simulated infected samples. By spiking variable WBC concentrations (50, 80, 100, 120, 150, 200, 250, and 300 WBCs/mm^3^) in effluent, we were able to train and validate our cell counter algorithm; samples spiked with concentrations of 100 WBCs/mm^3^ and above were considered “infected.” Using our prototype device, we captured 100 images of each spiked concentration sample as it flowed through our disposable chamber, using a peristaltic pump to simulate the cycler. These cell counts are then converted to concentrations via our counts processing algorithm.

### Image analysis and image processing

All computations for detecting fluid flow, image capture, and image analysis were performed locally on OpticLine’s Raspberry Pi 4 microprocessor, which runs on Raspberry Pi OS 2021. Once OpticLine detects fluid and takes images, these images are then analysed by a custom Python 3.7 script. Cell count outputs are saved locally on its SD storage.

### Counts processing algorithm cross validation training, testing, and summary statistics

The interquartile range data at each spiked concentration was split into *k* = 10 groups for the *k*-fold cross validation, in which one group is the test dataset for the regression model, and the other nine groups are the training datasets for the regression model. Within each training and testing group, concentration labels and algorithm counts were used to train and test the regression model. For a given *k*-fold, the linear regression parameters and predicted WBC concentrations were output. If a predicted WBC concentration was below zero, the prediction was set equal to zero, to normalise against any negative WBC concentration predictions. The score (*R*^2^) of the linear regression of the training data was also calculated. The data was heteroskedastic: the residuals of the regression model did not have constant variance and the image batch analysis outputs had varied ranges, with variability increasing as concentration increased. *R*^2^ was adjusted for heteroskedasticity via weighting by the conditional variance of the WBC predictions. This process was repeated for each *k*-fold. After the *k*-fold cross validation was complete, the average and standard deviation of regression parameters, *R*^2^, and WBC concentration predictions were calculated. The final regression model with parameters averaged over all *k*-fold iterations was then used to predict the concentration of image batch analysis count outputs from other clinically collected samples.

### Cell counting calibration and validation via manual counting

Using an image annotation tool^39^, one independent rater manually counted 347 images for visible cells. The rater started from the top left corner and scanned each image horizontally, finishing at the bottom right corner. The rater deemed darker circular or line-like shapes smaller than background artifacts and with distinct outlines to be cells. The tool presented a variable number of images per concentrations of 0, 10, 50, 100, 200, and 300 WBCs/mm^3^ to the rater. The rater’s counts were compared to algorithm counts to optimise sensitivity and specificity of the image batch analysis algorithm.

### Spiked effluent sample preparation

The simulated peritonitis samples (effluent samples with baseline WBC concentrations of ≤ 10 WBCs/mm^3^ and spiked final concentrations of approximately 100 WBCs/mm^3^) were created by determining the amount of isolated PBMCs to add to a given volume of the effluent sample. A baseline measurement of the effluent was first determined by a standard lab cell counter, the Cellometer Auto 2000 (Nexcelom Biosciences). Then, we drew blood from informed and consented healthy volunteers and isolated PBMCs using a Ficoll gradient density centrifugation^40^. We resuspended a counted number of PBMCs in a known volume of healthy patient effluent to produce concentrations of WBCs in effluent. We spiked multiple concentrations to train our algorithm.

### Confounder experiments sample preparation

Healthy effluent was placed in a 37 °C water bath. Aliquots were cooled to respective temperatures verified with a lab-grade infrared temperature gun. PBMCs were added to the aliquots and run through one metre of standard PD tubing and the OpticLine viewing chamber (our benchtop setup), in which they were imaged by the OpticLine clamp. Fluid flow was powered by a peristaltic pump, with flow rates mimicking those of a standard Baxter PD cycler.

### Statistical hypothesis testing

Wilcoxon signed-rank tests (with continuity correction), in addition to Pearson correlation coefficients, were used to identify least statistically significant differences between algorithm and manual counts by comparing *p*-values for image batch analysis parameter optimisation. To directly evaluate the measurements from our image batch analysis algorithm using the counts processing algorithm training data, Welch's *t*-tests (with Bonferroni correction) were used to compare normal (10, 50 WBCs/mm^3^) and infection-indicating (100 WBCs/mm^3^) algorithm outputs (α = .05). A Kruskal–Wallis test and post-hoc Dunn’s test with Bonferroni correction was used to evaluate statistically significant differences among groups of WBC concentration predictions for high concentration, added whole blood, and fluid flow confounder experiments, and a Mann–Whitney U test was used for temperature comparisons (α = .05). Shapiro–Wilk tests were used to test for normality before performing non-parametric tests (α = .05).

### Development of market research and human factors user studies

We conducted a 3–6 month revision process on a pilot usability study (Supplementary Note 5) to create two distinct “market research” and “human factors” studies using Qualtrics questionnaires. We conducted a literature review on best practices^41–43^, medical device adoption^44^, questionnaire readability^45^, demographics^46,47^, and patient-reported outcome measures as well as dialysis-focused questionnaire studies^48,49^. Both studies were approved by the Institutional Review Board of Stanford University (IRB-53127, IRB-60390) and were performed in accordance with the relevant guidelines and regulations. Our market research study was an anonymous online 10-min questionnaire administered from April to June 2021 to past, present, and prospective PD patients and caretakers, and also nephrologists, nurses, and researchers working with PD. We adapted PROMIS® (Patient-Reported Outcomes Measurement Information System) questions to further centre around the experiences of key stakeholders, modifying questions from PROMIS® Item Bank v.1.0 – General Life Satisfaction and PROMIS® Parent Proxy Item Bank v2.0 – Anxiety^50^ to ask specifically about PD experience and feelings. The questionnaire began with questions on OpticLine followed by PD patient history and care background questions. We gathered optional standard demographics information. We collected consent and assent without documentation to uphold anonymity. Participants completed a five-item feedback questionnaire after the main questionnaire. Recruitment methods included distribution through the National Kidney Foundation, displaying a QR code to the questionnaire on conference presentations and our website, as well as word-of-mouth. Our human factors study recruited paediatric caretakers from Lucile Packard Children’s Hospital to collect device setup times and user feedback. Study recruitment occurred between February–December 2021. Those with treating relationships with patients approached potential participants at or before dialysis clinic or at telehealth appointments with recruitment materials approved by the Institutional Review Board of Stanford University. Study team members obtained informed consent and assent at the dialysis clinic. Before or after the patient’s appointment, a chart review was conducted to collect relevant patient history.

## Supplementary Information

Supplementary Information.

## Data Availability

The main data supporting the findings of this study are available within the Article and its Supplementary Information. The raw data generated in this study are available from the corresponding author upon reasonable request, and pending licensing and patent status.

## Code availability

All source code for image analysis is available from the corresponding author upon reasonable request, and pending licensing and patent status.
