# Supplementary material for: Utilising low-cost, easy-to-use microscopy techniques for early peritonitis infection screening in peritoneal dialysis patients
Source: Sci Rep. 2022 Aug 18;12:14046. doi: 10.1038/s41598-022-18380-9 (PMC9388639; doi:10.1038/s41598-022-18380-9)
Supplement: Supplementary file 1 — Supplementary Information. [file 41598_2022_18380_MOESM1_ESM.pdf]

Supplementary Note 1. Frequency of OpticLine use.

We intend for OpticLine to be used during every PD session, although we have received feedback from stakeholders that weekly use may be preferable to increase compliance and decrease consumable costs. However, we cannot make a strong recommendation on OpticLine's optimal frequency of use, from both a measurement accuracy and compliance standpoint, without further clinical studies. We predict that a new chamber will be used for every PD session to prevent contamination and buildup that would cause optical interference, although we have confirmed that the same chamber can be used for multiple weeks at a time nonconsecutively and for ten days consecutively without any data capture discrepancies (Supplementary Fig. 2).

Supplementary Note 2. OpticLine use without disposable across Fresenius PD tubing set.

While many of our experiments used the standard Baxter PD tubing set, the Fresenius PD tubing set can also be used. In fact, the Fresenius tubing set has a transparent bubble in its drainage line. We directly imaged across this bubble using the OpticLine clamp without the disposable and learnt that the focal plane of our ball lens lies inside the thick bubble material, preventing us from acquiring quantifiable images of flowing cells. We replaced the ball lens with a 140x zoom compound lens. To do this, we swapped the standard Raspberry Pi V2 camera and printed circuit board with the Raspberry Pi HQ printed circuit board, which could then be attached to any off-the-shelf microscope lens via a C-mount. We then acquired a 140x zoom microscope lens and attached it to OpticLine directly. This new lens with a longer back focal length of 100 mm allowed us to image across the thick Fresenius tubing bubble material and acquire quantifiable images. We determined the number of cells in the viewing window per concentration and tested for statistically significant differences across concentrations (Supplementary Fig. 3). Similar to our regression analysis performed with the OpticLine spherical lens and disposable, we can eventually translate these counts to concentrations if we choose to take more images across several patient samples and timepoints to train a new prediction model with this new set-up.

Supplementary Note 3. Hough transform cell counting method.

One of the techniques we tested before developing the present image batch analysis algorithm was a common functionality termed the Hough transform (OpenCV 2021), which generally searches for circles within an image (Supplementary Fig. 5). This open source function from many third parties follows all of the contours of the image once a mask is produced. It then draws circles of variable sizes, as defined in the input parameters, along every contour over the entire image. If these circles overlap over a region in significant amounts, the function deems these regions as circle centres and outputs their centroids. Because cells are moving at high speeds across these frames, and because we are limited by the shutter speed of OpticLine's camera, many of our images contain cells that are not round but rather elliptical or even linear. We learnt that the Hough over-counted some regions of the images because the function has a high sensitivity. During validation, we realised that these regions consisted primarily of noise from turbulence associated with higher cell counts. This produced a larger dynamic range that helped the device discern variable concentrations with statistical differences. We were satisfied that the device was able to discern different concentrations of cells with the Hough but were dissatisfied that it wasn't only counting cells, but rather turbidity of the fluid associated with higher cell counts. Therefore, we pursued the present image batch analysis algorithm to have a higher specificity for cells. We also performed the classifications of WBC concentration predictions using the Hough algorithm count outputs (Supplementary Fig. 5). Interestingly, even though the Hough algorithm had slightly better training performance (R^2 was 0.94 vs. 0.93 for the current image batch analysis algorithm), the predictions using the Hough-trained linear regression model and count outputs resulted in a greater false positive rate (0.08), false negative rate (0.21), and lower accuracy (0.87), which further supports the development of our in-house and novel image batch analysis algorithm.

Supplementary Note 4. Cell detection performance in context of peritonitis screening.

When quantifying the performance of our image batch analysis algorithm for cell detection by comparing to manual cell counts, precision and accuracy tended to increase with increasing concentration (up to 73% and 31%, respectively), while lower concentrations had greater sensitivity (up to 45%). Having greater sensitivity at lower concentrations is helpful for our infection screening purposes so that we do not undercount, and thus underpredict WBC concentration, falsely classifying potentially infection-indicating cases as healthy. On the other hand, having greater precision at higher concentrations means that we will be less likely to overpredict WBC concentration at already high concentrations. We recognize that these performance results are relatively low for cell detection. Our focus for our screening tool is to discern healthy effluent from those at risk for infection, so we prioritize how well we can predict WBC concentration for binary classification rather than specific cell detection. See Figure 4 for classification accuracy results.

Supplementary Note 5. Nested cross-validation approach to training/validation of computational pipeline.

We performed the image batch analysis algorithm with 96 combinations of parameter values to output cell counts for images spanning six concentrations (healthy baseline to 120 WBCs/mm^3) in effluent from three patients. We focused on these lower concentrations to train our prediction model on critical ranges for infection classification, including multiple patient effluents to increase dynamic range when training and to make our model more robust to different patients. We randomly selected two different batches of five images out of twenty batches per concentration for each patient effluent sample for a total of 180 images.

Due to the linear nature of our cell-counting algorithm (Fig. 2b), we assumed a linear relationship between image cell counts and concentration. To select the parameter set that had the best concentration predictions, we performed k-fold (k=6) cross validation, holding out one set of five-image batches across the concentrations (Supplementary Fig. 8a). Within each cross-validation iteration, we trained 96 ordinary least squares linear regression models for each set of algorithm count outputs from different parameter combinations, after eliminating outliers by only training on the interquartile range (IQR) of the algorithm counts at each concentration in each cross-validation fold. In contrast to the present method, we regressed counts onto concentration ($counts = a*concentration + b$) so that our independent variable would be our "ground-truth" variable, and only corrected for negative concentration predictions on averages to minimise bias instead of variance. We calculated the R^2 on the IQR of the held-out test set of algorithm counts. We selected the fold-averaged linear regression model and parameter set that yielded the highest fold-averaged test R^2, which was 0.76 (Supplementary Table 3).

To evaluate the performance of this parameter-set selection method, we performed nested k-fold cross validation with an outer k=6 layer and an inner k=5 layer, so that one set of five-image batches were held out in each cross-validation layer (Supplementary Fig. 8b). Performing the same parameter-set selection method described previously in the inner cross-validation layer, we used the cross-validated linear regression model for the best parameter set to predict on the outer-layer IQR of the test set. The best parameter set resulting from the inner parameter-set selection method yielded the same parameter set in each outer cross-validation iteration, which was the same parameter set resulting from the single-layer cross validation (parameter-set selection). The average R^2 for the outer layer test sets was 0.77—similar to the final R^2 of the parameter-set selection method (Supplementary Table 3)—suggesting that our parameter-set selection method was robust.

Supplementary Note 5 (Continued):

We also had two independent raters each manually label the 180 total images for cells to evaluate how well our cell-counting algorithm performs compared to the ground truth of manual counts. We evaluated one rater's counts, the other rater's counts, and the average of their counts in place of the 96 parameter sets for both the single-layer parameter-set selection and nested cross-validation methods. For the single layer, the best manual count was one of the rater's, which had a fold-averaged test R^2 of 0.74, very similar to that obtained for our best parameter set (Supplementary Table 3). Although the best parameter set counts were generally high across concentrations (i.e., in the 100s for 0 WBCs/mm^3), they had the highest correlation with the best rater's counts as well as the raters' average counts, suggesting that our algorithm performs similarly to human raters. However, when performing the nested cross validation, there was less consistency for best-performing algorithm count set, and the average outer-layer test R^2 was 0.70, lower than that obtained from evaluating our algorithm counts (Supplementary Table 3). This suggests that while the parameter-set selection method and correlations demonstrated similarity between the manual and algorithm counts, we have less confidence in the single layer with the manual counts, and thus our algorithm achieves better performance than even human raters.

When using the single-layer fold-averaged regression model for the best-performing parameter set to predict on the effluent test samples, we found that the model under-predicted WBC concentrations more than the current model and was less able to classify the effluent samples into non-infection and infection-caution. We then tried performing the parameter-set selection and nested cross-validation pipelines on our original set of 100 images for six concentrations (healthy baseline to 300 WBCs/mm^3) of one patient's effluent. While this yielded a new best parameter set with more realistic counts (i.e. in the 10s-20s for 0 WBCs/mm^3) and higher parameter-set selection and nested cross-validation R^2 values (0.93 and 0.92, respectively), the concentration predictions were still under-predicted, resulting in a greater number of false negatives than our current model. When identifying what the linear regression model would be with our current algorithm counts from the parameter-set selection method described here, the false negative rate decreased, but the false positive rate increased. Although we aim to minimise the false negative rate, we decided to continue developing the current prediction model that had the best balance between sensitivity and specificity. We also believe that our current parameter-set selection method based on comparison to human raters' counts is more intuitive for the purposes of our publication.

Supplementary Note 5 (Continued):

While the approach of selecting the best parameter set by implementing the linear regression training within the selection process and using nested cross validation to evaluate the parameter-set selection method yielded promising results, we hypothesise that ultimately, there was so much variability among the algorithm counts compared to our training set size that it was difficult to obtain robust regression models when testing on counts from new images. Improving our counting algorithm against non-cell artifacts and increasing our training set size could yield better prediction performance for models resulting from the nested cross-validation approach.

Supplementary Note 6. Pilot market research and human factors study.

We conducted a pilot human factors study approved by the Institutional Review Board at Stanford to collect device feedback from our key stakeholders as soon as possible within our development timeline. Our team of physicians and engineers developed a 15-minute anonymous online questionnaire using Google Forms that was administered from December 2019 to March 2020 to PD past, present and prospective patients and caretakers, and also professionals working with PD, which includes nephrologists, nurses, and researchers. The questionnaire asks PD background questions, such as number of peritonitis episodes and how long it takes to set up PD, before leading into questions about three different design prototypes we were considering at the time, which included the present design. For participants recruited at Lucile Packard Children's Hospital, we additionally collected setup times of 3D-printed, non-functioning prototypes. There were six current and past PD patient and caretaker and 19 professional respondents, and the majority of the latter group believed OpticLine would reduce the time from infection to treatment (74%), patients' worries and fear (79%), and their own stress and workload (63%).

Supplementary Note 7. Additional screening metrics.

In clinical use, OpticLine would determine the screening result using thousands of images from multiple drains throughout the PD session. In our benchtop experiments, we created test infection samples and took the number of images that would be taken in a single drain (n=100). Thus, the present results demonstrate that we can achieve accurate screenings even from a single drain's worth of data, assuming that the test samples represent the average effluent fluid content from an entire PD session. Further clinical testing can indicate other summary statistics such as the mean or maximum WBC concentration prediction to provide additional information for screening. While the standard of care is just one WBC count at the end of a PD session, OpticLine tracks changes in WBC concentration throughout a PD session; novelly, our device may be able to notify patients of potential infection before the end of their PD session.

Supplementary Note 8. Spectrophotometry cell counting method.

Our original solution focused on developing a spectrophotometric approach to detecting the concentration of WBCs in peritoneal dialysis (PD) patients' effluent fluid as an early indication of peritonitis (Supplementary Fig. 11). Our initial experimentation to measure the optical absorbance of WBCs using the NanoDrop 2000 determined that the peak absorbance was at 265 nm, so we built the OpticLine prototype to measure the optical absorbance of our fluid samples at this wavelength. We tested our spectrophotometer works-like prototype using a range of WBC concentrations diluted in Dulbecco's phosphate-buffered saline (DPBS), mock effluent fluid, and PD patient effluent fluid. OpticLine measurements of mock effluent with added WBCs resulted in a significant difference between infected (132 WBCs/mm^3) and non-infectious WBC levels (10 WBCs/ mm^3) (Supplementary Fig. 11). However, when we tested PD effluent, we discovered that one of the four patients' effluent fluid used in the study confounded our results, preventing the OpticLine spectrophotometer prototype from being able to distinguish between infected and non-infected WBC concentrations. We concluded that spectrophotometry alone is not an optimal method for detecting peritonitis in PD patients due to the variability in fluid absorbance that may occur not only across patients, but also between PD sessions for a single patient. Although spectrophotometry used alone could lead to variable measurements, the technology could be used in tandem with microscopy (the focus of our current WBC detection method) to allow for more accurate measurements with a multi-cheque system.

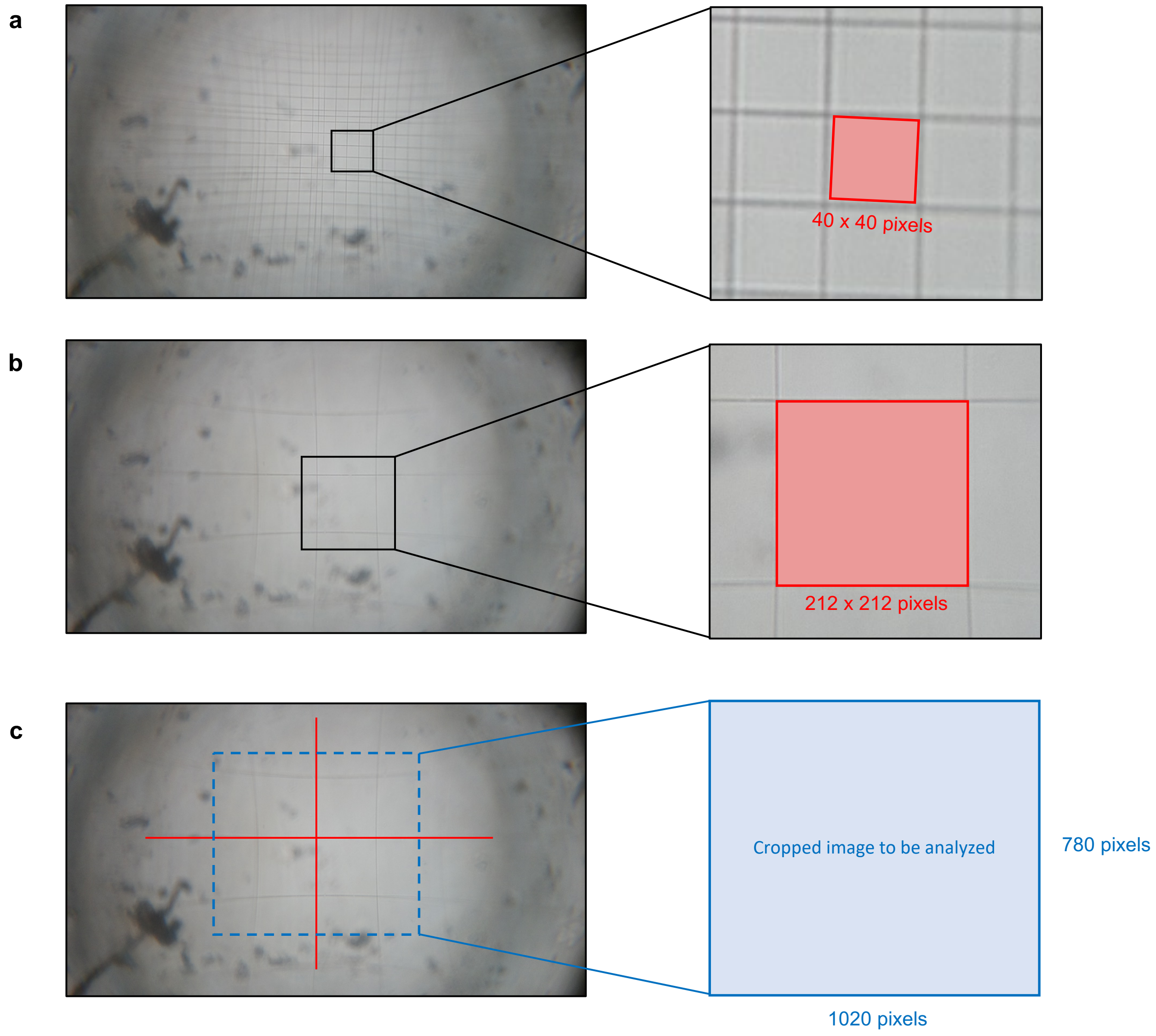

Supplementary Figure 1. OpticLine resolution and field of view validation. a, Raw image of OpticLine viewing window with hemacytometer grid, with region of interest pop-out focused on the smaller grid, consisting of squares 50×50 μm, which translate to 40×40 pixels in the image taken. **b,** Raw image of OpticLine viewing window with hemacytometer grid, with region of interest pop-out focused on the larger grid, consisting of squares 250×250 μm, which translate to 212×212 pixels in the image taken. **c,** Raw image of OpticLine viewing window with larger hemacytometer grid to depict where the gridlines (red) are out of focus for OpticLine's ball lens. When images are taken, they are then cropped to 780×1020 pixels (blue with pop-out) to ensure that analysis occurs only within OpticLine's field of view.

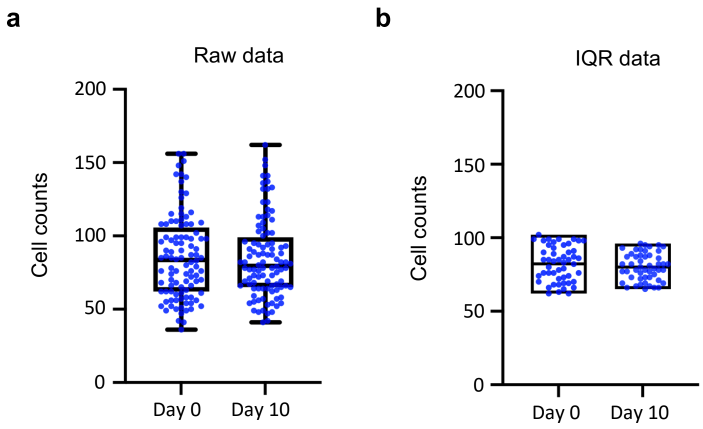

Supplementary Figure 2. Disposable chamber reusability. a, Batches of images (*n*=100) were taken at Day 0 with effluent fluid resuspended with 100 WBCs/mm^3. This spiked fluid was then discarded. For the next eight hours, a peristatic pump pumped healthy effluent fluid (0-10 WBCs/mm^3) through the PD tubing and OpticLine for 10 minutes every hour. This was repeated for 10 days. At day 10, batches of images (*n*=100) were taken with effluent fluid resuspended with 100 WBCs/mm^3. The images were analysed and their cell counts are plotted. **b,** To limit noise, raw counts were filtered by taking the interquartile range (IQR), only keeping the middle 50% of the counts. There is no significant differences in the counts between day 0 and day 10.

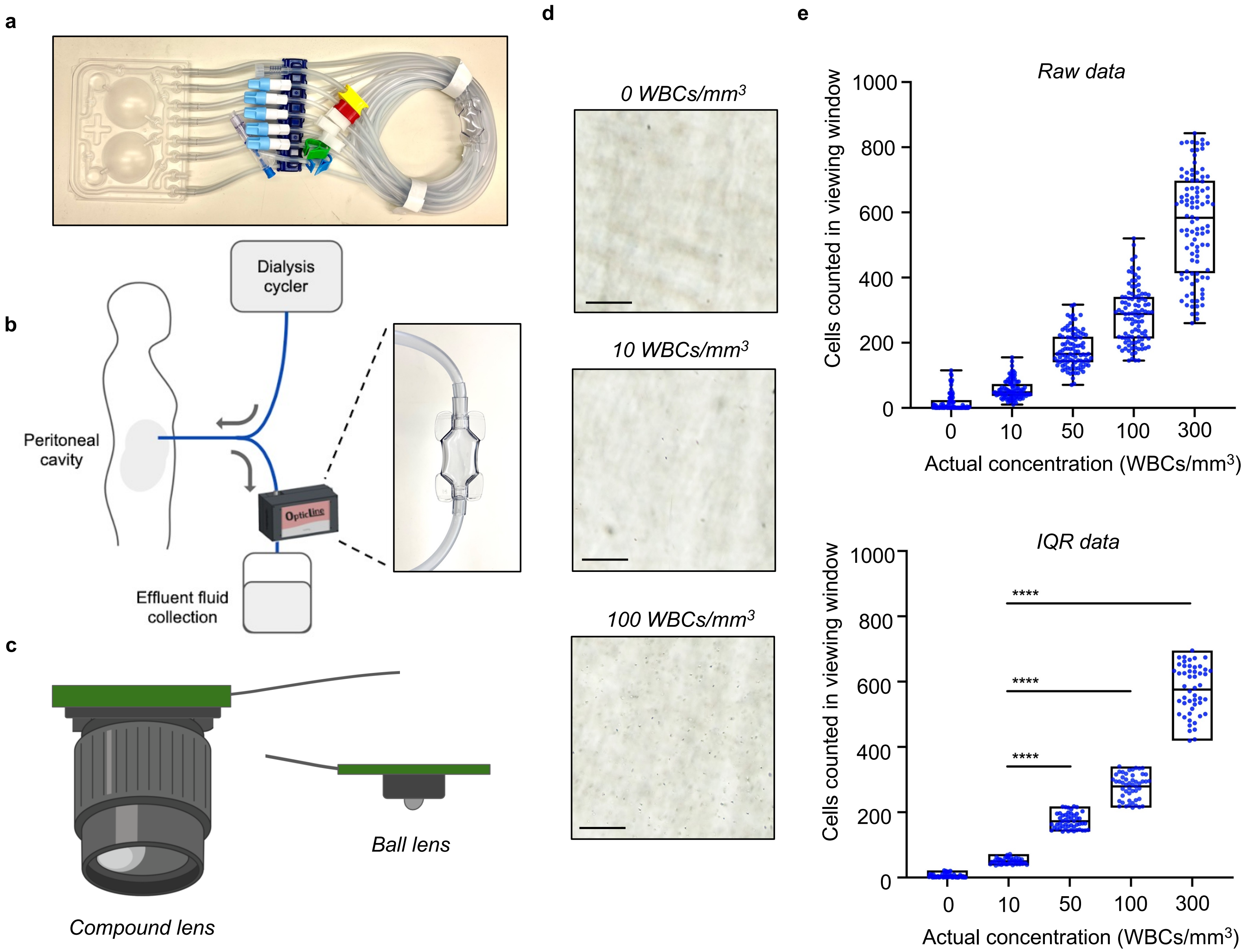

Supplementary Figure 3. OpticLine operating with compound lens. a, Standard Fresenius peritoneal dialysis tubing set. **b,** Diagram of OpticLine connected to Fresenius tubing set so its optical hardware aligns with the transparent plastic bubble from the tubing set. Created with Onshape.com. **c,** Diagram depicting the ball lens and the microscope lens mounted to OpticLine's printed circuit board. Created with BioRender.com. **d,** Images taken by OpticLine and the compound lens across the Fresenius tubing bubble at multiple concentrations of WBCs in effluent fluid flowing at 300 mL/min. Scale bar represents 200 microns. **e,** Batches of images (n=100) were taken of effluent fluid resuspended at multiple concentrations of WBCs across the Fresenius tubing bubble while fluid was flowing at 300mL/min. The images were analysed by OpticLine and their cell counts are plotted before regression analysis. To limit noise, raw counts were filtered by taking the interquartile range (IQR), only keeping the middle 50% of the counts. **** = p<0.001 determined by two-tailed Student's t-tests.

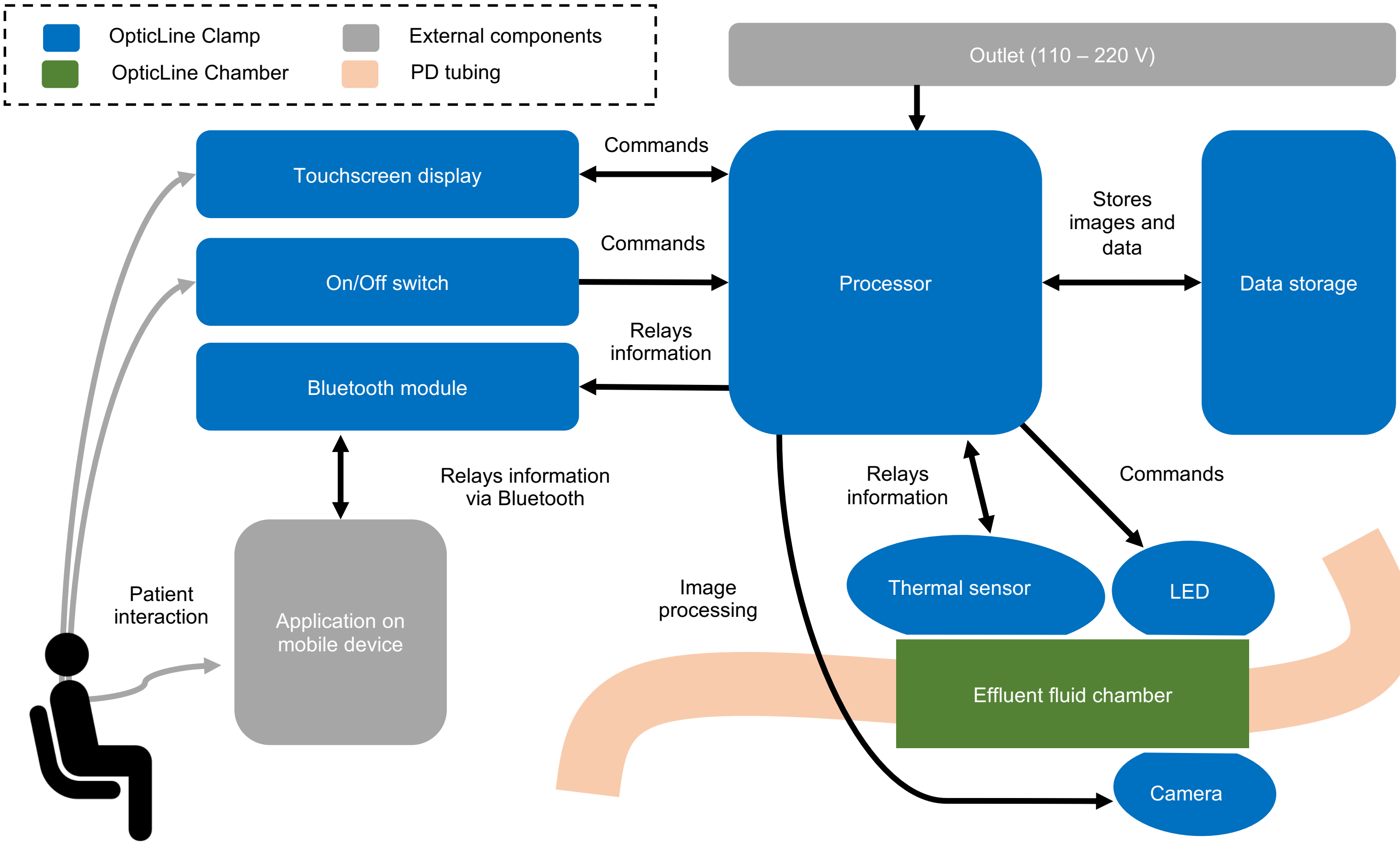

Supplementary Figure 4. OpticLine block diagram. A block diagram depicting the connectivity and interactions of all components in OpticLine, as explained further in **Methods**.

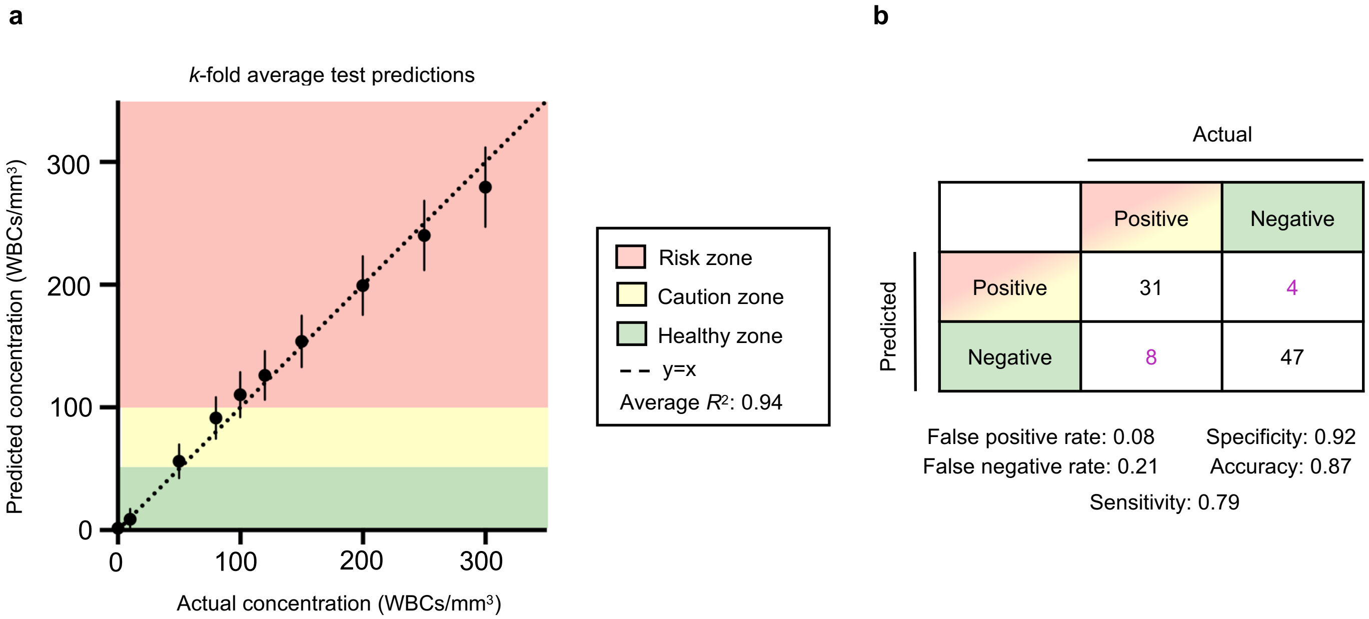

Supplementary Figure 5. Hough algorithm output summary. a, Graph of the *k*-fold average test predictions across *k*=10 iterations of the cross validation vs. actual concentrations for images across *n*=18 effluent samples. Error bars are s.d. The predicted and actual WBC concentrations were similar, falling close to one-to-one correlation as represented by the *y=x* line (dotted line) with average R^2 across the *k*=10 iterations of 0.94. **b,** Confusion matrix of binary classification using Hough transform algorithm on all test effluent samples with 50 WBCs/mm^3 as the threshold for “positive” for infection-caution (yellow, red zones) for predicted WBC concentrations above, and “negative” for infection-caution (green zone) for predicted WBC concentrations below. Samples of effluent (*n*=90) were collected from peritoneal dialysis patients. Baseline WBC concentrations were evaluated for all collected samples using Cellometer Auto 2000 (Nexcelom Biosciences). Some of the samples with healthy baselines (0-10 WBCs/mm^3) were spiked with WBCs to a concentration of 100 WBCs/ mm^3. False positive rate, false negative rate, specificity, sensitivity, and accuracy were calculated.

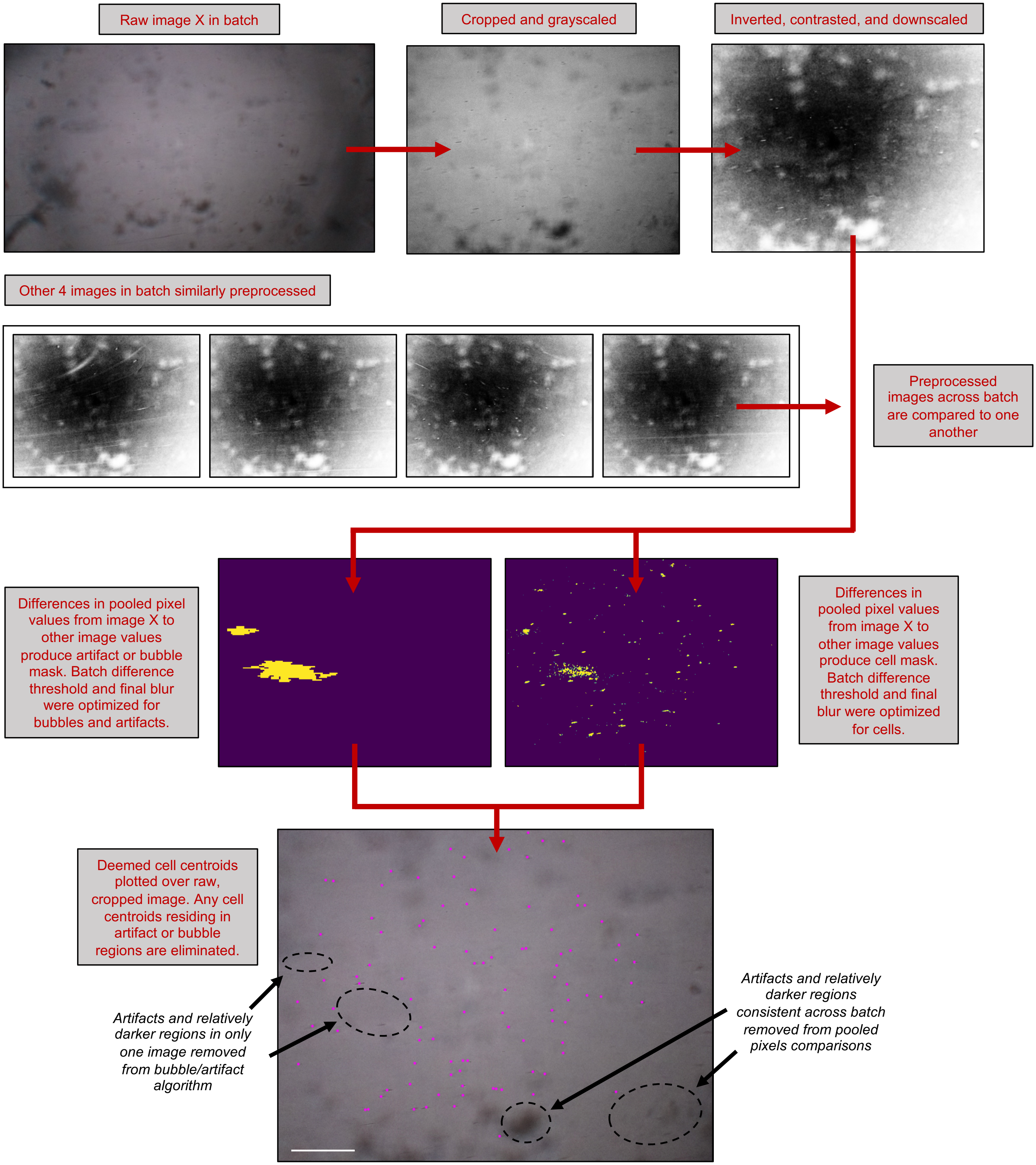

Supplementary Figure 6. OpticLine image batch analysis workflow with artifact removal examples. Diagram of how a batch of five images taken by OpticLine is preprocessed through filters (cropped, grayscaled, inverted, contrasted, and downscaled), then compared to one another through pooled pixel analysis, producing a cell and artifact mask per image. Final cell centroids are shown with examples of artifacts being removed. Scale bar represents 200 microns.

a

Concentration (cells/mm^3)	Sample Size	Precision	Accuracy	Sensitivity
0	64	20.96 (18.74)	17.32 (21.56)	44.78 (58.72)
10	80	25.40 (18.63)	19.82 (17.54)	41.98 (32.47)
50	81	39.71 (15.41)	20.53 (10.44)	32.61 (20.01)
100	49	48.52 (11.58)	26.69 (11.09)	38.44 (18.42)
200	11	73.89 (11.87)	31.17 (10.14)	36.99 (16.29)
300	62	73.01 (14.01)	31.29 (12.28)	37.66 (17.88)
	**347**	**41.23 (20.66)**	**22.90 (5.59)**	**38.88 (4.68)**

b

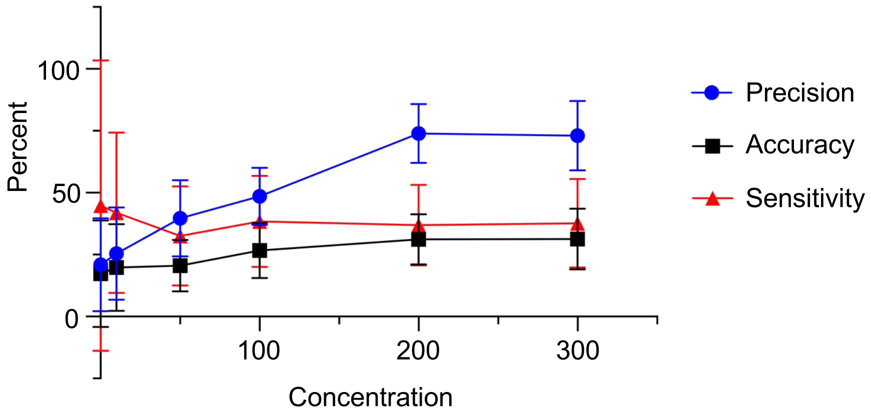

Supplementary Figure 7. Quantitative performance metrics for image batch analysis algorithm. The single-rater manual cell counts for *n*=347 images across white blood cell concentrations of 0-300 cells/mm^3 were compared to the algorithm's counts. If the coordinates of a manually-counted cell was within 30 pixels of the coordinates of an algorithm-counted cell in an image, the cell was deemed as a true positive (TP). False positives (FP) were counted as having an algorithm-counted cell without a nearby manually-counted cell, and false negatives (FN) were counted as having a manually-counted cell without a nearby algorithm-counted cell. Precision was calculated as TP/(TP+FP), accuracy was calculated as TP/(TP+FP+FN), and sensitivity was calculated as TP/(TP+FN). **a,** Table of image sample size, and mean precision, accuracy, and sensitivity per each concentration. s.d. is in parentheses. The bottom row indicates the total number of images (*n*=347), and the weighted average precision, accuracy, and sensitivity across all concentrations. Weighted s.d. is in parentheses. Weights were determined by the proportion of images at each concentration. **b,** Graph of mean precision (blue circles), accuracy (black squares), and sensitivity (red triangles) versus concentration. Error bars are s.d.

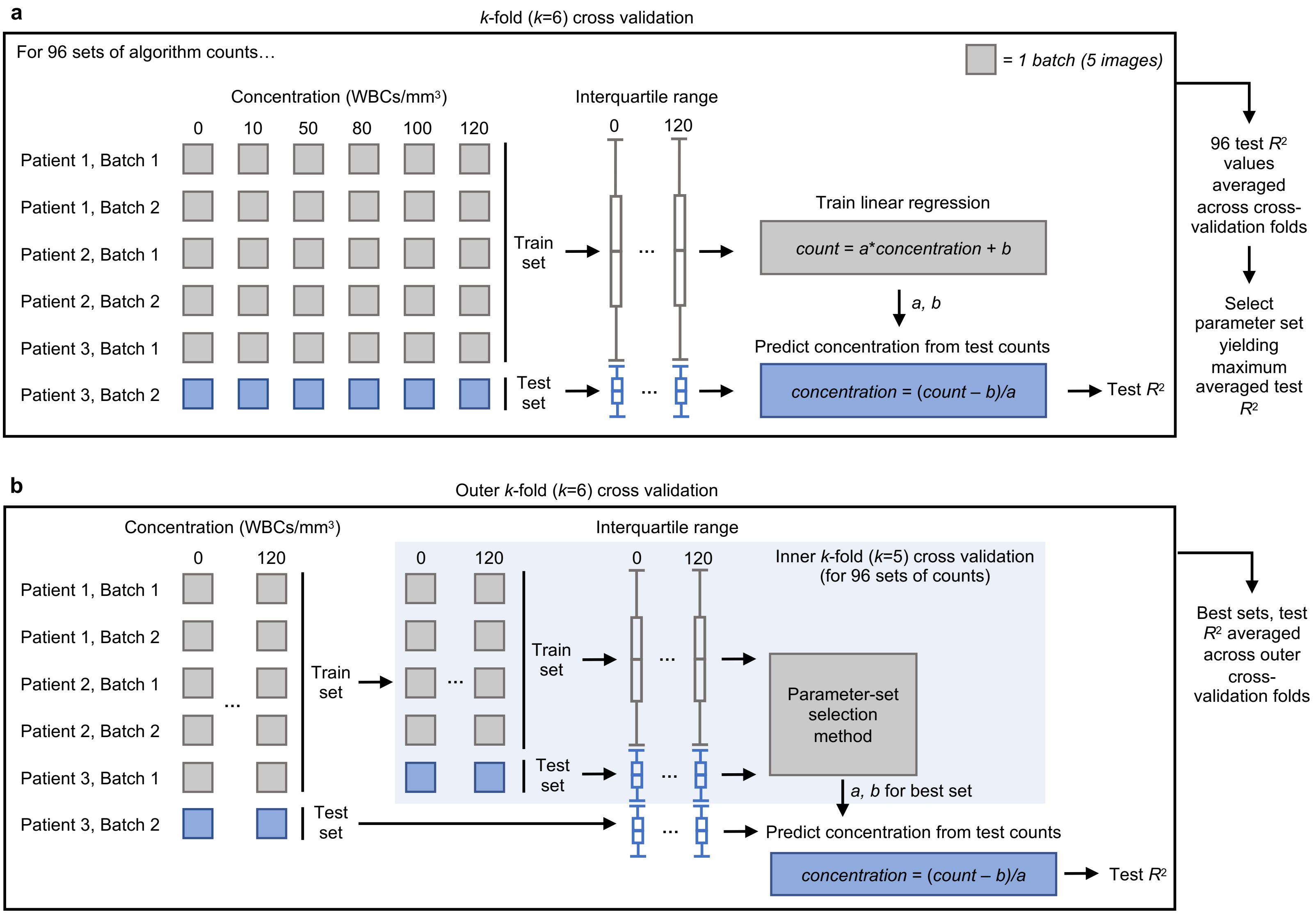

Supplementary Figure 8. Parameter-selection and nested cross validation pipelines. **a,** Parameter-selection pipeline. For each set of algorithm counts resulting from 96 different combinations of algorithm parameters, *k*-fold (*k*=6) cross validation was performed over two batches per three different patients, holding out one batch as a test set. The interquartile range was taken for both the training and test sets to eliminate outliers before training a simple linear regression model or predicting concentration from test counts using the linear regression parameters (*a, b*), respectively. After cross validation, all test R^2 values were averaged across the folds for each parameter set, and the fold-averaged linear regression model from the parameter set that yielded the maximum fold-averaged R^2 was used to predict concentrations of new effluent samples. **b**, Nested cross validation pipeline to evaluate parameter-selection method. *k*-fold (*k*=6) cross validation was also performed, but the training set was further divided into inner training and test sets for *k*=5 cross validation, performing the same parameter-selection method described in **a**. The fold-averaged regression model associated with the maximum fold-averaged R^2 was used to predict concentration on the outer-layer test set. For each outer-cross validation iteration, the parameter sets with the highest inner fold-averaged test R^2 values (“best sets”) and the outer fold-averaged test R^2 value were calculated to assess parameter-selection performance.

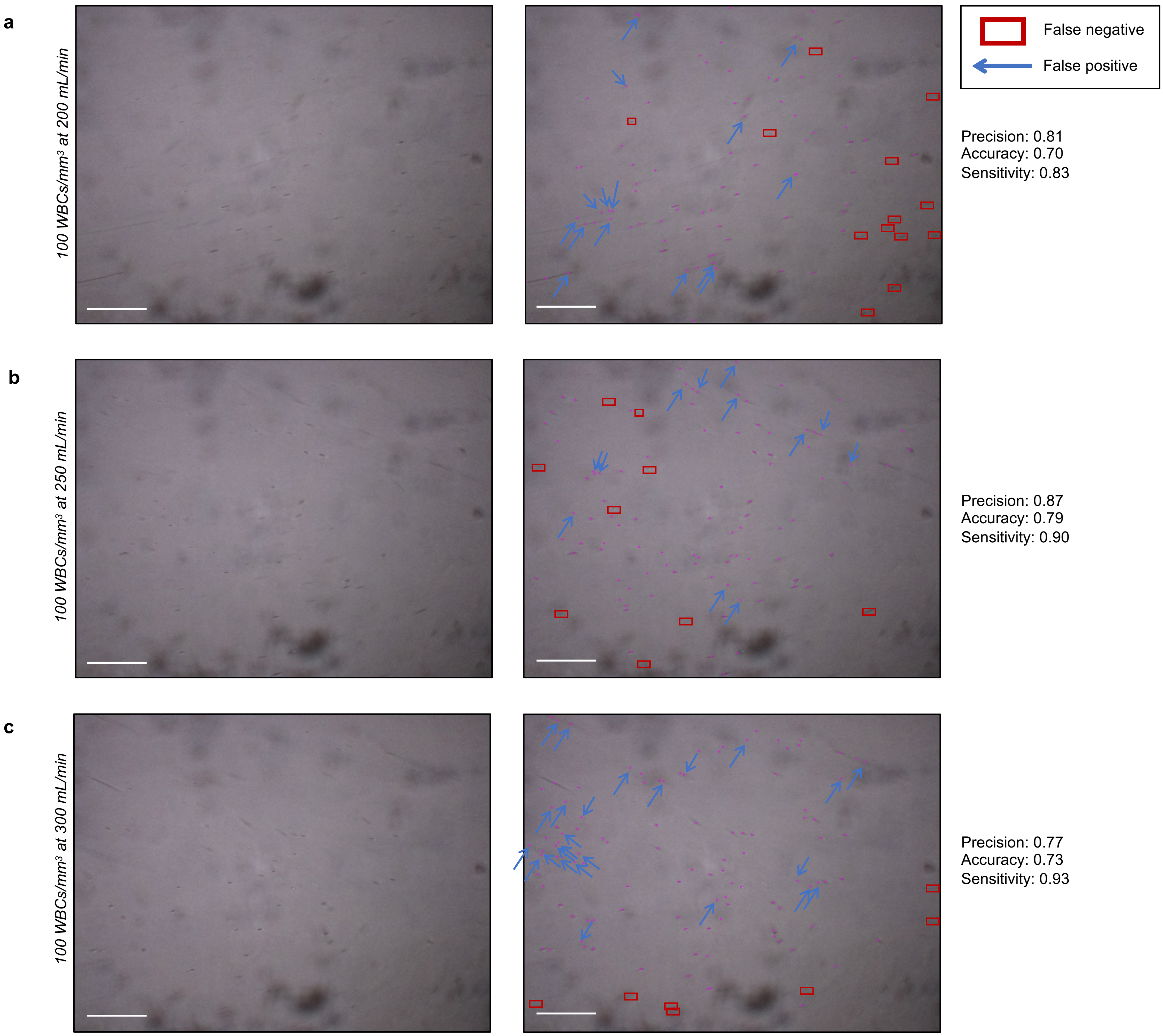

Supplementary Figure 9. Quantitative performance metrics for image batch analysis algorithm for sample images at various flow rates. a, b, c, Left: Cropped raw image taken by OpticLine for 100 WBCs/mm^3 at various flow rates (200 mL/min, 250 mL/min, 300 mL/min). Right: Cropped raw image overlaid with algorithm-deemed cell centroids (purple), with blue arrows depicting false positive (FP) cells and red boxes depicting false negative (FN) cells compared to manually-counted cells, alongside the precision, accuracy, and sensitivity per image. Precision was calculated as true positive (TP)/(TP+FP), accuracy was calculated as TP/(TP+FP+FN), and sensitivity was calculated as TP/(TP+FN). Scale bar represents 200 microns.

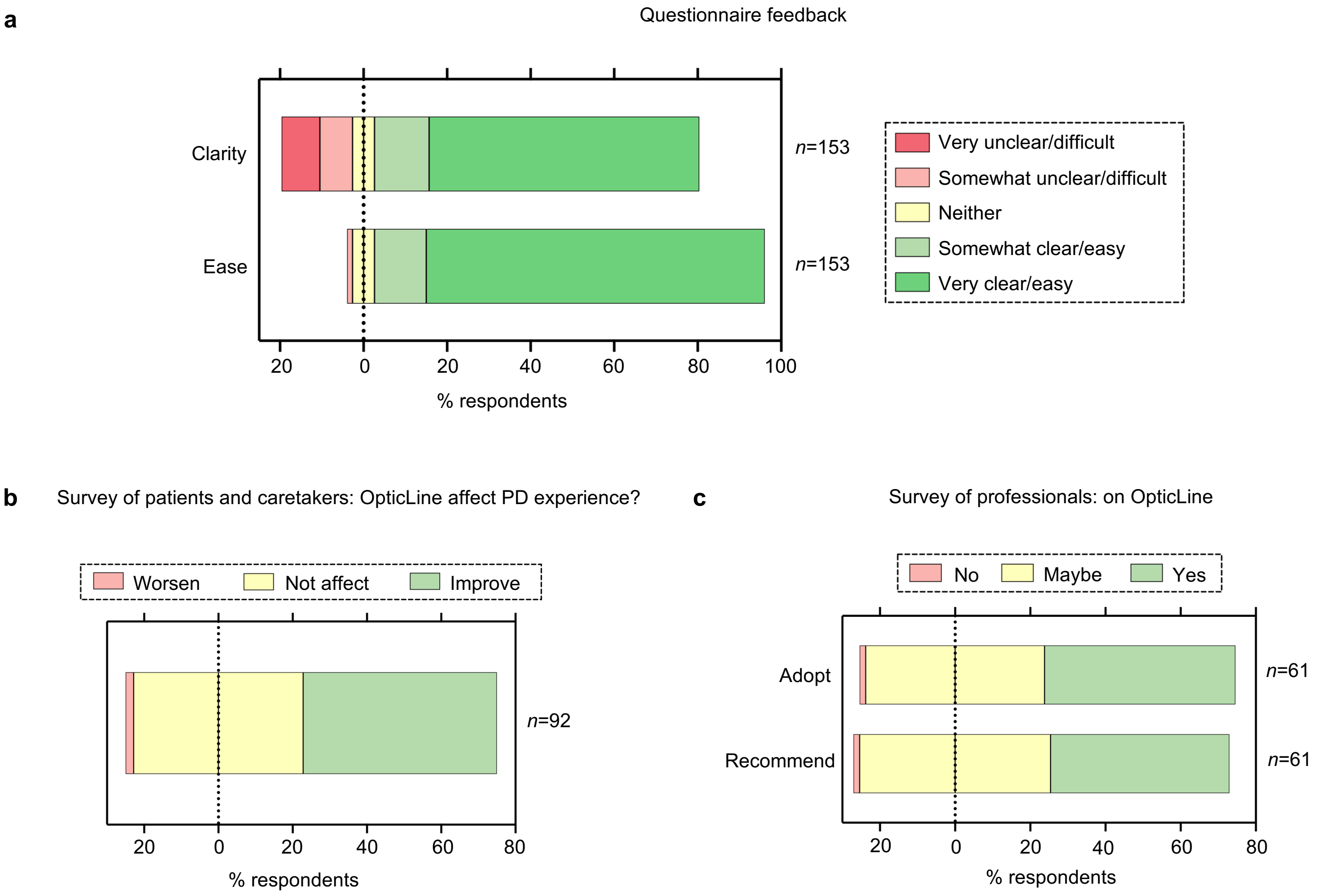

Supplementary Figure 10. Market research questionnaire feedback and three-point rankings for opinions on OpticLine. Percentage of respondents for each question shown on the *x*-axis and number of respondents (*n*) shown on the right *y*-axis. **a,** Five-point Likert scale results for feedback on the market research questionnaire from all respondents (*N*=153) ranking clarity of the questions (top) and ease of completion (bottom). Likert scale was very unclear or difficult (left, red) to very clear or easy (right, green), respectively. **b,** All patient and caretaker respondents (*n*=92) were asked if they think OpticLine would worsen (left, red), not affect (middle, yellow), or improve (right, green) their experience with peritoneal dialysis (PD). **c,** All professional respondents (*n*=61) responded with no (left, red), maybe (middle, yellow), or yes (right, green) for being in favour of adopting the device into standard clinical practise for PD and recommending the device to their PD patients.

a

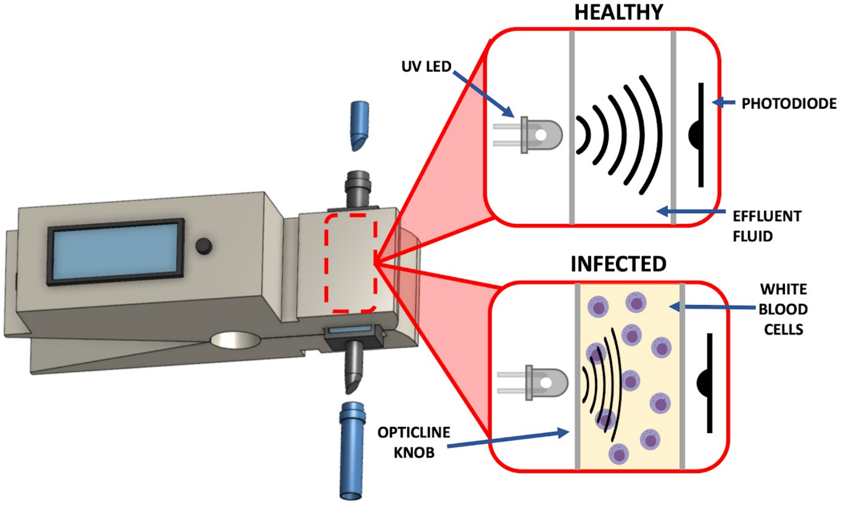

b

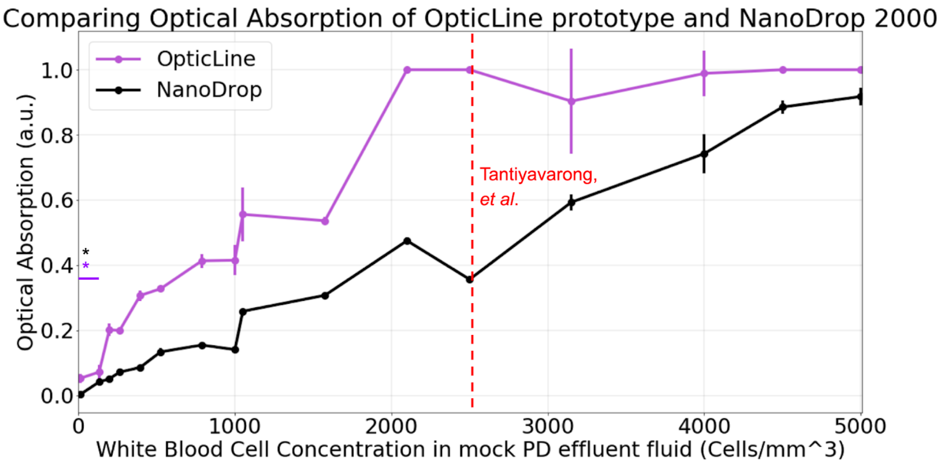

Supplementary Figure 11. Spectrophotometry approach to quantify white blood cells (WBCs). a, Experimental setup for OpticLine spectrophotometry prototype. The device fits in-line with the standard peritoneal dialysis setup and measures WBCs in real time using an ultraviolet LED where a photodiode measures the optical absorbance. Created with Onshape.com and BioRender.com. **b,** Optical absorption data of WBCs at different concentrations using a NanoDrop 2000 (black) and OpticLine's spectrophotometer prototype (purple). The graph shows the peak optical absorption data, which occurred at 265 nm. WBCs were diluted in mock effluent fluid and measurements were taken in triplicate (three technical replicates). Error bars s.d. Statistically significant difference (*$p<0.05$) was found between the baseline WBC concentration of 10 WBCs/mm^3 and peritonitis-level WBC concentration of 132 WBCs/mm^3. The red bar shows the average WBC concentration measured for the patients who arrived in clinic after the onset of peritonitis.

Supplementary Table 1. Complete bill of materials for OpticLine reusable clamp with cost-breakdown.

Part	Source	Unit price (USD)*
Raspberry Pi 4 (4 GB)	Link	55
SD storage (32 GB)	Link	8
Custom Fritzing printed wiring board		10
Power supply 5.1V@ 3A	Link	8
Pi camera V2.1	Link	30
Neutral white 4000K hemispheric high power LED	Link	8
Thermal sensor from Adafruit	Link	16
3.5" Nextion touchscreen display	Link	33
Spherical 140x lens	Link	2
PLA plastic for device embodiment and printing		10
Additional components (capacitors, resistors, wiring, hinges, gasket, nuts, screws, etc.)		20
TOTAL		200

*Unit price per single unit.

Supplementary Table 2. Complete bill of materials for OpticLine disposable chamber with cost-breakdown.

Part	Unit price (USD)*
Injection molding estimate for chamber body (medical grade polycarbonate)	0.42
Optical-grade polycarbonate sheet for chamber windows	0.04
TOTAL	0.46

*Unit price per 3000 units. Price for one prototype unit printed via stereolithography (SLA) with two glass coverslips totals to $40.

Supplementary Table 3. R^2 values for single-layer cross validation and outer-layer in the nested cross validation pipeline for algorithm and manual counts for 180 images.

Algorithm counts	Parameter-selection (single-layer)	0.76
	Outer-layer	0.77
Manual counts	Rater-selection (single-layer)	0.74
	Outer-layer	0.70

Supplementary Table 4. Market research questionnaire respondent breakdown (*N*=171; *N*=153 valid responses).

Category	*n* of respondents (% of total)
Nephrologist, researcher, nurse, or other professional who works with PD	61 (35.7%)
Current PD patient	50 (29.2%)
Past PD patient	29 (17.0%)
None*	12 (7.0%)
None but I know someone who does PD*	6 (3.5%)
Current PD caretaker	4 (2.3%)
Prospective PD patient on hemodialysis (HD) or another renal replacement therapy (RRT)	3 (1.8%)
Prospective PD patient not yet on any renal replacement therapy (RRT)	3 (1.8%)
Past PD caretaker	2 (1.2%)
Prospective PD caretaker	1 (0.6%)

*Did not complete questionnaire.
“PD” stands for peritoneal dialysis.

Supplementary Table 5. Market research respondent characteristics for all patients (*n*=92).

Category	*n* of respondents (% of total)*
Age (years)	
0-18	1 (1.1%)
19-44	17 (18.5%)
45-64	51 (55.4%)
65-74	14 (15.2%)
75+	9 (9.8%)
Sex	
Male	31 (33.7%)
Female	61 (66.3%)
Ethnicity	
Hispanic	4 (4.4%)
Non-Hispanic	82 (90.1%)
Unknown	1 (1.1%)
Race	
White	62 (67.4%)
Black/African American	15 (16.3%)
American Indian or Alaska Native	0 (0%)
Asian	7 (7.6%)
Native Hawaiian or Pacific Islander	1 (1.1%)
Other or Multiracial	2 (2.2%)
Unknown	1 (1.1%)
Location**	
West	20 (22.7%)
Midwest	17 (19.3%)
Southwest	3 (3.4%)
Northeast	18 (20.5%)
Southeast	29 (33.0%)
International	1 (1.1%)

*All other responses if percentage does not add up to 100% for each category were "prefer not to disclose."
**First five geographical locations refer to those in the U.S. Percentage calculated from those who reported a state or not living in the U.S. (*n*=88); *n*=90 reported living in the U.S.

Supplementary Table 6. Market research respondent characteristics for all professionals (n=61).

Category	n of respondents (% of total)*
Age (years)	
19-44	22 (36.1%)
45-64	28 (45.9%)
65-74	7 (11.5%)
75+	1 (1.6%)
Sex	
Male	22 (36.1%)
Female	38 (62.3%)
Ethnicity	
Hispanic	2 (3.3%)
Non-Hispanic	53 (86.9%)
Unknown	1 (1.6%)
Race	
White	32 (52.5%)
Black/African American	4 (6.6%)
American Indian or Alaska Native	0 (0%)
Asian	15 (24.6%)
Native Hawaiian or Pacific Islander	1 (1.6%)
Other or Multiracial	2 (3.3%)
Unknown	1 (1.6%)
Location**	
West	14 (25.0%)
Midwest	10 (17.9%)
Southwest	5 (8.9%)
Northeast	11 (19.6%)
Southeast	8 (14.3%)
International	8 (14.3%)

*All other responses if percentage does not add up to 100% for each category were “prefer not to disclose.”
**First five geographical locations refer to those in the U.S. Percentage calculated from those who reported a state or not living in the U.S. (n=56); n=51 reported living in the U.S.

Supplementary Table 7. Peritoneal dialysis history for current and past patient respondents in market research study (*n*=85).*

Category	*n* of respondents (% of total)
Time on peritoneal dialysis (years)	
<0.5	9 (11.0%)
0.5-1	21 (25.6%)
2-5	44 (53.7%)
6-10	6 (7.3%)
11-15	1 (1.2%)
>15	1 (1.2%)
Peritonitis rates	
Times diagnosed	
0	47 (55.3%)
1	22 (25.9%)
2	2 (2.4%)
3	6 (7.1%)
4	4 (4.7%)
5+	4 (4.7%)
Times hospitalized	
0	58 (71.6%)
1	15 (18.5%)
2	1 (1.2%)
3	6 (7.4%)
4	1 (1.2%)
5+	0 (0%)

*Caretaker respondents were asked to respond on behalf of their patient.

Supplementary Table 8. Pediatric patient history of caretakers participating in the human factors study (*N*=9).

Category	*n* of respondents (% of total)
Age (years)	
0-9	1 (11.1%)
10-18	8 (88.9%)
Sex	
Male	4 (44.4%)
Female	5 (55.6%)
Ethnicity	
Hispanic	5 (55.6%)
Non-Hispanic White	3 (33.3%)
Native Hawaiian	1 (11.1%)
No. of confirmed peritonitis episodes	
0	7 (77.8%)
1+	2 (22.2%)
No. of past presumed contamination episodes	
0	8 (88.9%)
1+	1 (11.1%)
No. of hospitalizations for peritonitis	
0	7 (77.8%)
1+	2 (22.2%)
No. of hospitalizations for non-peritonitis PD complication	
0	6 (66.7%)
1+	3 (33.3%)

"No." stands for number.
"PD" stands for peritoneal dialysis.

References:

1. Hemocytometer square size • Hemocytometer. *Hemocytometer* https://www.hemocytometer.org/hemocytometer-square-size/ (2013).

2. OpenCV: Hough Circle Transform. https://docs.opencv.org/3.4/d4/d70/tutorial_hough_circle.html.

3. Tantiyavarong, P., Traitanon, O., Chuengsaman, P., Patumanond, J. & Tasanarong, A. Dialysate White Blood Cell Change after Initial Antibiotic Treatment Represented the Patterns of Response in Peritoneal Dialysis-Related Peritonitis. *Int. J. Nephrol.* **2016**, (2016).
